# Pituitary Adenylate Cyclase-Activating Polypeptide (PACAP) Protects Striatal Cells and Improves Motor Function in Huntington’s Disease Models: Role of PAC1 Receptor

**DOI:** 10.3389/fphar.2021.797541

**Published:** 2022-01-28

**Authors:** Irene Solés-Tarrés, Núria Cabezas-Llobet, Benjamin Lefranc, Jérôme Leprince, Jordi Alberch, David Vaudry, Xavier Xifró

**Affiliations:** ^1^ New Therapeutic Targets Group, Department of Medical Science, Faculty of Medicine, University of Girona, Girona, Spain; ^2^ Laboratory of Neuronal and Neuroendocrine Communication and Differentiation, Neuropeptides, Neuronal Death and Cell Plasticity Team, UNIROUEN, Inserm, Normandie University, Rouen, France; ^3^ Regional Cell Imaging Platform of Normandy (PRIMACEN), UNIROUEN, Normandie University, Rouen, France; ^4^ Departament de Biomedicina, Institut de Neurociències, Facultat de Medicina, Universitat de Barcelona, Barcelona, Spain; ^5^ Institut D’Investigacions Biomèdiques August Pi i Sunyer (IDIBAPS), Barcelona, Spain; ^6^ Centro de Investigación Biomédica en Red Sobre Enfermedades Neurodegenerativas (CIBERNED), Madrid, Spain

**Keywords:** PACAP, PAC1R, neurodegenerative diseases, Huntington’s disease, neuroprotection, BDNF

## Abstract

Huntington’s disease (HD) is a hereditary neurodegenerative disorder caused by the expression of mutant huntingtin (mHtt). One of the main features of HD is the degeneration of the striatum that leads to motor discoordination. Pituitary adenylate cyclase-activating polypeptide (PACAP) is a neuropeptide that acts through three receptors named PAC1R, VPAC1R, and VPAC2R. In the present study, we first investigated the effect of PACAP on STHdhQ7/Q7 and STHdhQ111/Q111 cells that express wild-type Htt with 7 and mHtt with 111 glutamines, respectively. Then we explored the capacity of PACAP to rescue motor symptoms in the R6/1, a murine model of HD. We found that PACAP treatment (10^–7^ M) for 24 h protects STHdhQ111/Q111 cells from mHtt-induced apoptosis. This effect is associated with an increase in PAC1R transcription, phosphorylation of ERK and Akt, and an increase of intracellular c-fos, egr1, CBP, and BDNF protein content. Moreover, the use of pharmacological inhibitors revealed that activation of ERK and Akt mediates these antiapoptotic and neurotrophic effects of PACAP. To find out PAC1R implication, we treated STHdh cells with vasoactive intestinal peptide (VIP), which exhibits equal affinity for VPAC1R and VPAC2R, but lower affinity for PAC1R, in contrast to PACAP which has same affinity for the three receptors. VIP reduced cleaved caspase-3 protein level, without promoting the expression of c-fos, egr1, CBP, and the neurotrophin BDNF. We next measured the protein level of PACAP receptors in the striatum and cortex of R6/1 mice. We observed a specific reduction of PAC1R at the onset of motor symptoms. Importantly, the intranasal administration of PACAP to R6/1 animals restored the motor function and increased the striatal levels of PAC1R, CBP, and BDNF. In conclusion, PACAP exerts antiapoptotic and neurotrophic effects in striatal neurons mainly through PAC1R. This effect in HD striatum allows the recovery of motor function and point out PAC1R as a therapeutic target for treatment of HD.

## Introduction

Huntington’s disease (HD) is an autosomal inherited neurodegenerative disorder caused by an expanded CAG codon repeat in the *huntingtin* gene (The Huntington’s Disease Collaborative Research Group, 1993). The resulting expression of the mutant huntingtin protein (mHtt) elicits a loss of gamma-aminobutyric acid (GABA)ergic striatal projection medium spiny neurons (Vonsattel and DiFiglia, 1998) leading to a striatal atrophy, which is correlated with clinical motor impairment, the most characteristic symptom in HD (Guo et al., 2012). Two early pathologic events underlie HD striatal cell death: transcriptional dysregulation and brain derived neurotrophic factor (BDNF) deprivation (Cha, 2007). The presence of mHtt impairs the levels and activity of different transcriptional-related proteins in the striatum, such as cyclic-adenosine monophosphate (cAMP) response element-binding protein (CREB)-binding protein (CBP). It has been found that CBP depletion directly contributes to mHtt-induced neurotoxicity (Jiang et al., 2006). This is because CBP dysfunction or altered levels result in a down-regulation of CREB/CBP-dependent genes involved in neuronal survival such as c-fos, egr1 (Wyttenbach, 2001), and of the neurotrophin BDNF (McCampbell, 2000; McCampbell and Fischbeck, 2001). Reduced BDNF protein levels have been found in the striatum and cortex of multiple HD mouse models (Luthi-Carter, 2000; Zuccato et al., 2001; Gines et al., 2003b; Giralt et al., 2009) and diminished striatal BDNF levels have been associated with HD clinical motor disorders (Ferrer et al., 2000; Zuccato and Cattaneo, 2007). Therefore, compounds able to restore altered transcriptional profile and BDNF levels in the striatum of HD could be pharmacological strategies to prevent neuronal loss and motor symptomatology.

Pituitary adenylate cyclase-activating polypeptide (PACAP) is an endogenous neuropeptide that acts as neurotransmitter, neuromodulator and neurotrophic factor in the brain (Vaudry et al., 2009). PACAP actions are mediated through three subtypes of G protein-coupled receptors: the PAC1R, the VPAC1R and the VPAC2R (Vaudry et al., 2009). Importantly, PAC1R is the major binding site for PACAP, while VPAC receptors have equal affinity for both PACAP and vasoactive intestinal peptide (VIP) (Harmar et al., 2012). In cultured neurons, PACAP counteracts apoptotic processes through the stimulation of several transduction mechanisms such as the cAMP/extracellular signal-regulated kinases (ERK)/CREB and Phosphoinositide 3-kinases (PI3K)/Protein kinase B (Akt) pathways (Bhave and Hoffman, 2003), and the induction of the transcription of neuroprotective genes such as c-fos and Bcl-2 (Falluel-Morel et al., 2004; Aubert et al., 2006), leading to the blockage of caspase-3 activation (Dejda et al., 2008). In addition to its direct antiapoptotic effect, PACAP also exerts neuroprotection by increasing Bdnf transcription (Frechilla et al., 2001; Rat et al., 2011). PACAP neuroprotective capacity has also been demonstrated in models of Parkinson’s disease (PD) induced by 1-methyl-4-phenylpyridinium ion (MPP^+^); (Chung et al., 2005; Deguil et al., 2010), 6-hydroxy-dopamine (6-OHDA) (Takei et al., 1998; Reglodi et al., 2004) or rotenone (Wang et al., 2005) and in models of Alzheimer’s disease (AD) induced by beta-amyloid protein (Frechilla et al., 2001; Onoue et al., 2002; Rat et al., 2011; Brown et al., 2013). Furthermore, PACAP not only prevents neurodegeneration but also improves cognitive functions (Solés-Tarrés et al., 2020). In HD, the role of PACAP has been poorly explored so far although we have recently described that PACAP administration prevents hippocampal-dependent cognitive decline in the transgenic R6/1 mouse model of HD (Cabezas-Llobet et al., 2018). Moreover, PACAP has been demonstrated to protect striatal neurons against quinolinic acid-induced cell death (Tamás et al., 2006). However, the ability of PACAP to prevent mHtt-mediated striatal death and associated motor symptomatology has not been explored yet. Therefore, with the present study we investigated the capacity of PACAP to protect cultured striatal cells from mHtt-induced toxicity and the effect of PACAP intranasal administration on motor deficits in a transgenic HD mouse model.

## Materials and Methods

### STHdH Cell Line

Immortalized striatal knock-in cells stably expressing full-length wild-type (WT) Htt with 7 glutamines (STHdhQ7/Q7) or full-length mHtt with 111 glutamines (STHdhQ111/Q111) were generated from WT HdhQ7/Q7 and homozygous HdhQ111/Q111 mice (Gines et al., 2003a). Cells were grown in Dulbecco’s modified Eagle’s medium (DMEM; Sigma-Aldrich, Saint Louis, MO, United States), supplemented with 10% fetal bovine serum (FBS, HyClone Laboratories, GE Healthcare, Chicago, IL, United States), 1% streptomycin-penicillin (Lonza, Basel, Switzerland), 2 mM L-glutamine (Lonza), 1 mM sodium pyruvate (Lonza) and maintained at 33°C, as previously described (Gines et al., 2003b). Cells were deprived from FBS to investigate the effects of PACAP on cell survival or apoptosis.

### Pharmacological Treatments in STHdh Cells

**FIGURE 1:**
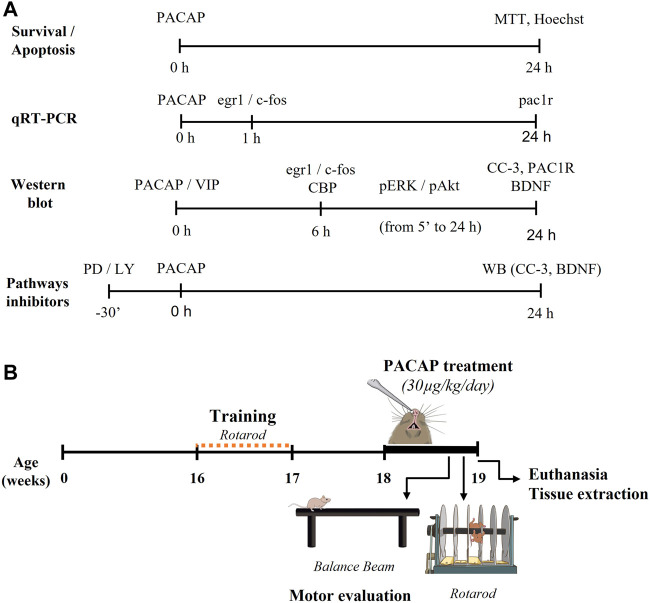
Experimental design of the study. **(A)** Methodology performed to determine cell viability and molecular changes in STHdh cells after the pharmacological treatments. STHdhQ7/Q7 and STHdhQ111/Q111 cells were treated with PBS as vehicle, PACAP (10^–7^ M) and/or VIP (10^–7^ M) for the appropriate number of hours. Then, we studied cell viability by MTT assay and Hoechst stain, the gene expression by qRT-PCR, and the protein levels by Western blotting. To inhibit ERK and PI3K-Akt pathways, cells were exposed to PD98059 (25 µM) or LY294002 (10 µM), respectively, for 30 min before PACAP treatment. **(B)** Methodology performed to study the capacity of PACAP to improve the motor deficits of R6/1 HD mouse model. At the age of 18 weeks, mice were daily treated intranasally with PACAP (30 μg/kg/day) for 7 days. Motor function was evaluated after 5 days of treatment by the Balance Beam test and after 6 days by Rotarod test. At the 7th day of treatment, animals were sacrificed by cervical dislocation and the striatum was rapidly dissected out.

STHdhQ7/Q7 and STHdhQ111/Q111 cells were treated at 80% confluence with phosphate buffered saline (PBS) as a vehicle, PACAP38 (PACAP; 10^–7^ M) or VIP (10^–7^ M). Peptides were synthesized using solid phase strategy combined with the Fmoc chemistry methodology as previously described (Jolivel et al., 2009). The experimental design is summarized in Figure 1A. Briefly, to study cell viability and quantify apoptosis, cells were treated with PACAP for 24 h. To determine protein expression by Western blotting, cells were treated for 5 min to 24 h with PACAP or VIP. To determine gene expression by qRT-PCR, cells were treated with PACAP for 1 or 24 h. To inhibit ERK and PI3K-Akt pathways, STHdhQ7/Q7 and STHdhQ111/Q111 cells were exposed to PD98059 (25 µM) or LY294002 (10 μM, both from Sigma-Aldrich) 30 min prior to PACAP treatment.

### Cell Viability Assay

Cell viability was determined using a 3-(4,5-dimethyl-2-thiazolyl)-2,5-diphenyl-2 H-tetrazolium bromide (MTT) assay. STHdhQ7/Q7 and STHdhQ111/Q111 cells were seeded in 96-well plates in their corresponding growth medium. After treatment with vehicle or PACAP for 24 h, MTT (Sigma-Aldrich) was added to each well (0.5 mg/ml) and incubated for 1 h at 37°C. Subsequently the medium was sucked and dimethyl sulfoxide (DMSO; Sigma-Aldrich) was added to dissolve formazan resulting from MTT metabolism. Finally, the absorbance was measured in a microplate spectrophotometer (Benchmark Plus, BioRad, Hercules, CA, United States) at 570 nm. The assay was performed in triplicates and repeated at least four times.

### Quantification of Apoptosis

Quantification of apoptotic cell death was performed using the Hoechst 33,258 (Invitrogen, Carlsbad, CA, United States) nucleic acid stain as explained elsewhere (Anglada-Huguet et al., 2012). Briefly, STHdhQ7/Q7 and STHdhQ111/Q111 cells treated with vehicle or PACAP were fixed in 4% paraformaldehyde in PBS for 10 min, washed twice in PBS and stained with Hoechst 33,258 (1:10,000) for 5 min at room temperature. After washing twice with PBS, the coverslips were mounted with Mowiol-mounting media (Merck, Darmstadt, Germany). Nuclear DNA staining was observed with a fluorescence microscope (Olympus). Condensed or fragmented nuclei were considered apoptotic. At least 300 cells were evaluated for each condition in each independent experiment. All the analysis was performed in a blinded fashion.

### Quantitative Reverse Transcription Polymerase (qRT-PCR)

After 1 or 24 h of PACAP treatment, cells were washed with PBS, and then 1 ml of Qiazol (Qiagen, Hilden, Germany) was added. Total-RNA was isolated using the RNeasy mini kit (Qiagen) following the instructions provided by the manufacturer. cDNA was synthesized from 1 µg of total RNA at 25°C for 5 min, followed by 42°C for 60 min using a High Capacity Archive Kit (Applied Biosystems, Foster, United States). The reaction was terminated by heating at 70°C for 5 min and cDNA was diluted to 5 ng/μl. 1 μl was used to perform qRT-PCR. Gene expression levels of selected genes were assessed using a LightCycler^®^ 480 Real-time PCR System (Roche Basel, Switzerland) with LightCycler^®^ 480 SYBR Green I Master (Roche). Primers used for target genes are reported in Table 1 (all from Sigma-Aldrich). The PCR thermal-cycling program included an initial activation step at 95°C for 10 min followed by 40 cycles of a two-step PCR at 95°C for 15 s and 60°C for 30 s. All RT-PCR analyzes were run in triplicate in five independent experiments. Relative RNA expression levels were calculated using GAPDH as a control.

**TABLE 1:** Primer design.

PAC1R	Forward	CCC​TGA​CTG​CTC​TCC​TCC​TGC​TGC​CTA​T
	Reverse	CAG​GGC​AGC​TCA​CAA​GGA​CCA​TCT​CAC​C
EGR1	Forward	GGGAGCCGAGCGAACAA
	Reverse	GTC​TCC​ACC​AGC​GCC​TTC​T
C-FOS	Forward	GAT​GTT​CTC​GGG​TTT​CAA​CGC​G
	Reverse	ACG​TCG​GTA​GAA​TAA​GGA​AAG​GG
GAPDH	Forward	ATC​ATC​CCT​GCC​TCT​ACT​GG
	Reverse	GTC​AGG​TCC​ACC​ACT​GAC​AC

### Genetically Modified HD Mouse Model

R6/1 transgenic mice expressing exon-1 of mHtt were obtained from Jackson Laboratory (Bar Harbor, ME, United States) and maintained on a B6CBA background. Our R6/1 colony displayed 145 CAG repeats (Giralt et al., 2009). Mice were genotyped by polymerase chain reaction as described previously (Mangiarini et al., 1996). Wild-type littermate animals were used as the control group. All mice were male and were housed together in groups of mixed genotypes with access to food and water ad libitum. The colony room was kept at 19–22°C and 40–60% humidity, under a 12-h light/dark cycle. Experiments were conducted in a blind-coded manner with respect to genotype and treatment, and data were recorded for analysis by microchip mouse number.

### PACAP Administration in R6/1 Mice

Eighteen weeks old WT and R6/1 mice were treated intranasally (i.n.) with PBS as vehicle (VEH) or PACAP38 (PACAP; 30 μg/kg). Concentration of PACAP was decided based on previous studies (Cabezas-Llobet et al., 2018). Administration was daily performed for 7 days, and then motor assessment was carried out (Figure 1B). Finally, animals were sacrificed by cervical dislocation and the striatum and motor cortex were rapidly dissected out. Seven animals were used for each condition.

### Motor Assessment

#### Balance Beam

Animals’ balance was evaluated using the balance beam test measuring the ability of animals to cross a narrow beam. The beam consisted of a steel cylinder (50 cm) with a 15 mm-round diameter and divided in 5 cm frames. It was placed horizontally, above the bench surface with each end mounted on a narrow support. At the end of PACAP administration (19 weeks of age) animals were allowed to walk for 2 min along the beam, and the number of slips and the distance covered were measured.

#### Rotarod

Balance and motor coordination were evaluated using the rotarod apparatus. At the end of PACAP administration (19 weeks of age), animals were placed on a motorized cylinder (30 mm diameter) with a fixed rotation speed. The number of falls in a total of 60 s was recorded at the speeds of 16 and 24 rpm. Prior to these experiments, animals were trained for 3 days at the age of 16 weeks

### Protein Extraction and Western Blotting

In cell cultures, STHdhQ7/Q7 and STHdhQ111/Q111 cells treated with vehicle or PACAP were homogenized with ice-cold lysis buffer 1% Triton X-100, 10% glycerol, 50 mM Tris-HCl (pH 7.5), 10 mM EDTA and 150 mM NaCl supplemented with protease and phosphatase inhibitors (2 mM phenylmethylsulphonyl fluoride, 10 μg/μl aprotinin, 1 μg/μl leupeptin, 2 mM Na_3_VO_4_) (all from Sigma-Aldrich). WT and R6/1 were killed by cervical dislocation at the age of 8, 12, 20, and 30 weeks. R6/1 animals treated with vehicle or PACAP were sacrificed by cervical dislocation on the last day of motor assessment. In all cases, the brain was quickly removed, and motor cortex and striatum were dissected out and homogenized with the same ice-cold lysis buffer described above. In all cases, samples were centrifuged at 16,100 × *g* for 20 min at 4°C, and supernatants were collected. Protein concentration was determined using the Dc protein assay kit (Bio-Rad Laboratories). Then, protein extracts (10–30 μg) were resolved by 7–15% sodium dodecyl sulfate-polyacrylamide (Bio-Rad Laboratories) gel electrophoresis and transferred to nitrocellulose membranes (ThermoFisher Scientific, MA, United States). Blots were blocked in 5% non-fat powdered milk in Tris-buffered saline containing 0.1% Tween-20 (TBS-T, Sigma-Aldrich) solution for 1 h at room temperature. The membranes were then incubated overnight at 4°C with anti-PAC1R, anti-VPAC1R, anti-VPAC2R (all 1:500; Santa Cruz Biotechnology), anti-BDNF (1:1,000; Santa Cruz Biotechnology), anti-c-fos, anti-CBP (all 1:250; Santa Cruz Biotechnology), anti-cleaved caspase-3 (1:500; Cell Signaling Technology, Danvers, MA), anti-caspase-3, anti-egr1, anti-pERK1/2, anti-ERK1/2, anti-pAkt or anti-Akt (all 1:1,000; Cell Signaling Technology) primary antibodies. Loading control was performed by reprobing the membranes with anti-α-tubulin (1:50,000; Sigma-Aldrich) or anti-β-actin (1:1,000; Santa Cruz Biotechnology) primary antibodies depending on the molecular weight of the protein of interest. The use of both proteins as loading controls is widely accepted in STHdh cellular model (Gines et al., 2003a; Paoletti et al., 2008; Saavedra et al., 2010; Xifró et al., 2011; Leitman et al., 2014; Di Pardo et al., 2020; Huang et al., 2021; Ibrahim et al., 2021) while in R6/1 mouse model of HD, anti-α-tubulin is more commonly used (Creus-Muncunill et al., 2019; Cherubini et al., 2020; Alcalá-Vida et al., 2021). Membranes were washed in TBS-T and incubated with corresponding horseradish peroxidase-conjugated secondary antibodies (1:2,000; Promega, Madison, United States) for 1 h at 25°C. Immunoreactive bands were detected using Immobilon Western Chemiluminiscent HRP Substrate (Millipore Corporation, Billerica, United States) in a Bio-Rad ChemiDoc™ MP Imaging System (Bio-Rad Laboratories). Bands corresponding to the molecular weight of each protein were quantified using Image Lab Software (Bio-Rad Laboratories). Then, phosphorylation levels and protein expression of each subject was normalized to its own actin or tubulin levels.

### Statistical Analysis

**FIGURE 2:**
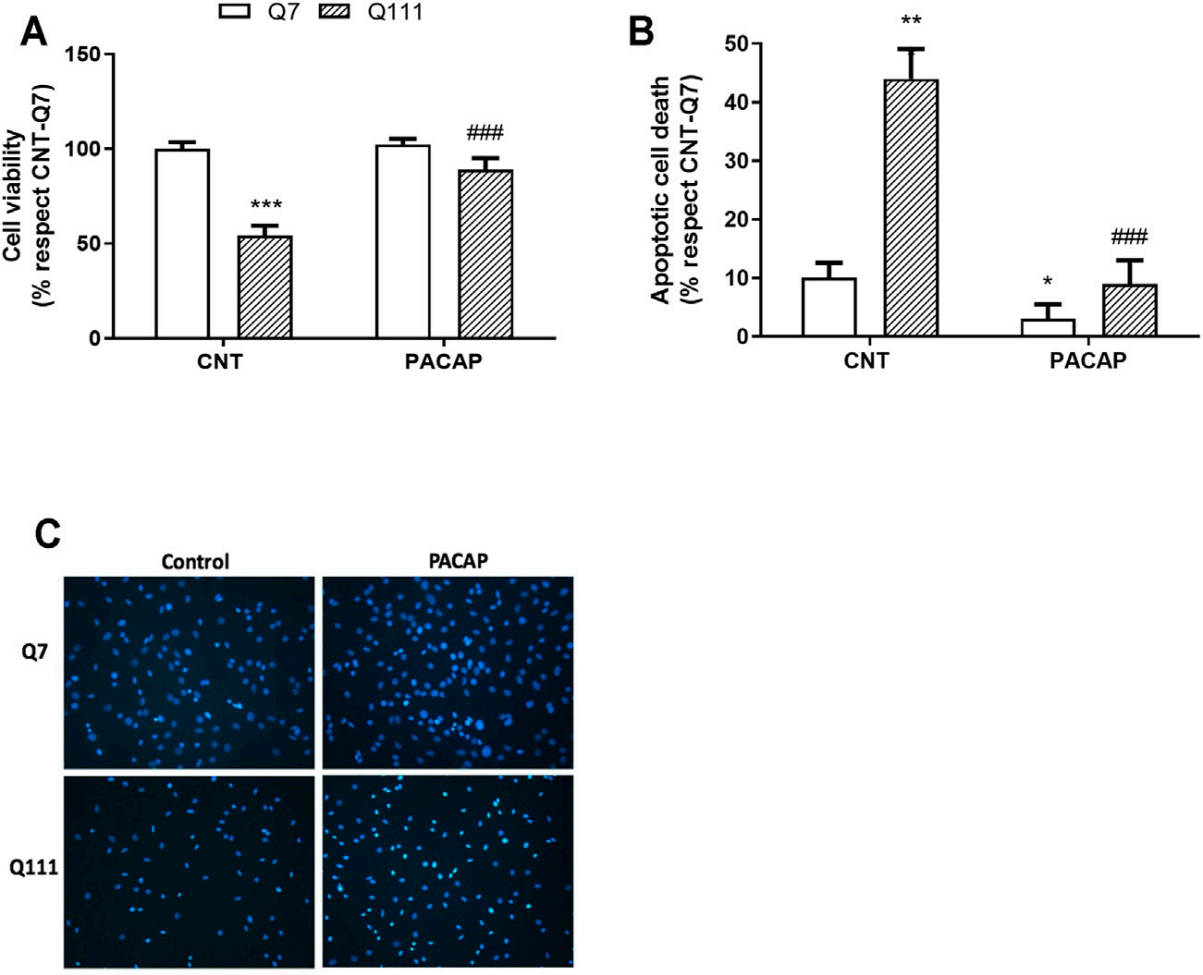
PACAP promotes cell viability in STHdhQ111/Q111 cells. STHdhQ7/Q7 (Q7) and STHdhQ111/Q111 (Q111) cells were treated with PBS (CNT) or PACAP (10^−7^ M) for 24 h. **(A)** Cell survival was analyzed by the MTT assay. Data are expressed as percentage respect to CNT-Q7 cells and results are the mean ± SEM (*n* = 4). **(B)** Apoptotic nuclei were counted using the Hoechst 33,258 staining. Results are expressed as the mean ± SEM (*n* = 4) of apoptotic nuclei with respect to the total nuclei in CNT-Q7 cells. **(C)** Representative images of Hoechst 33,258 staining are shown. Scale bar 20 µm. In all cases, data were analyzed by using two-way ANOVA followed by Tukey as a post-hoc test. *p < 0.05, **p < 0.01, and ***p < 0.001 as compared to CNT-Q7 cells; ###*p* < 0.001 as compared to CNT-Q111 cells.

**FIGURE 3:**
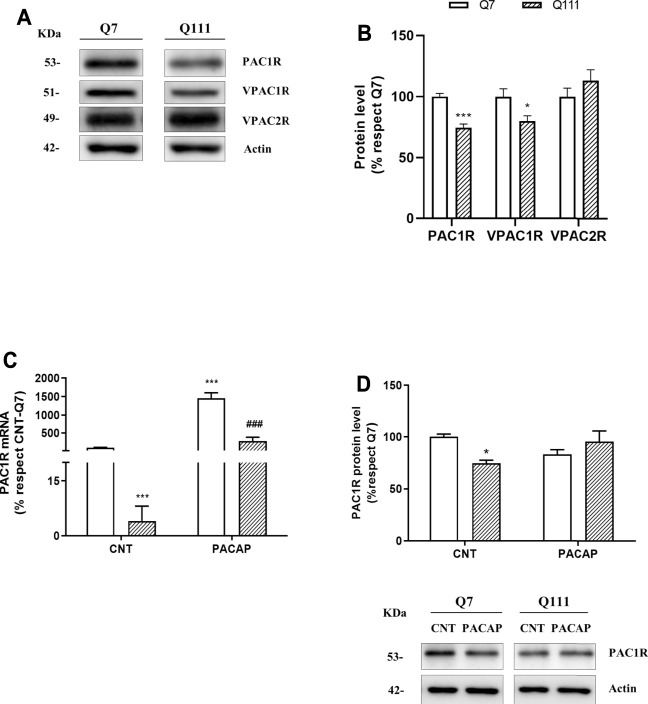
PAC1R is reduced in STHdhQ111/Q111 cells and the addition of PACAP increases its mRNA levels. Protein levels of PAC1R, VPAC1R, and VPAC2R were analyzed in STHdhQ7/Q7 (Q7) and STHdhQ111/Q111 (Q111) cells by Western blot. **(A)** Representative immunoblots are shown. Quantification of **(B)** PAC1R, VPAC1R, and VPAC2R was performed. The results (mean ± SEM; n = 5) were normalized to the loading control actin and are expressed as a percentage respect to Q7 cells. Then, STHdhQ7/Q7 (Q7) and STHdhQ111/Q111 (Q111) cells were treated with PBS (CNT) or PACAP (10-7M) for 24 hours **(C)** mRNA levels of PAC1R were analyzed using quantitative RT-PCR. The results (mean ± SEM; n = 5) were normalized to GAPDH and are expressed as a percentage respect to CNT-Q7 cells. **(D)** Protein levels of PAC1R were studied by Western blot. The results (mean ± SEM; n = 5) were normalized to the loading control actin and are expressed as a percentage respect to CNT-Q7 cells. Representative immunoblots are shown. In **(B)**, comparison of receptors’ protein levels between Q7 and Q111 was analyzed by using Student’s t-test. In **(C)** and **(D)**, data were analyzed by using two-way ANOVA followed by Tukey as post-hoc test. *p < 0.05 ***p < 0.001 as compared to CNT-Q7 cells; ###*p* < 0.001 as compared to CNT-Q111 cells.

**FIGURE 4:**
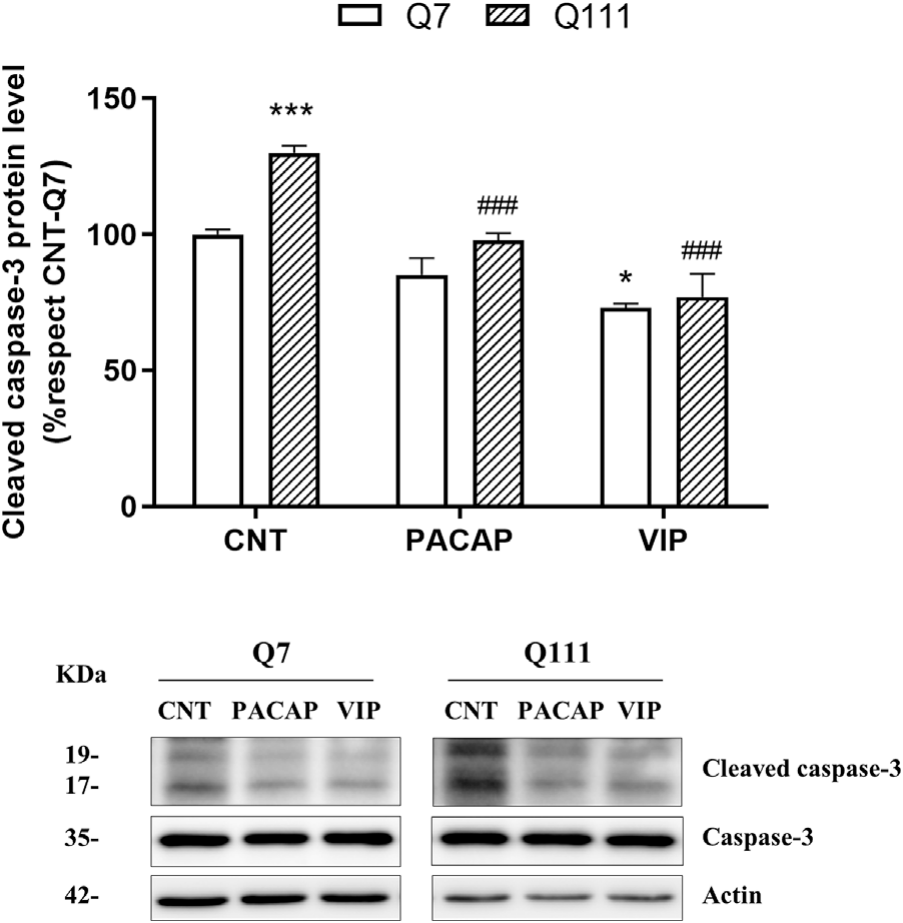
PACAP and VIP reduce apoptosis in STHdhQ111/Q111 cells. STHdhQ7/Q7 (Q7) and STHdhQ111/Q111 (Q111) cells were treated with PBS (CNT), PACAP (10^−7^ M) or VIP (10^−7^ M) for 24 h in a serum free media. Then, cleaved caspase-3 protein levels were measured by Western blot. As no changes were detected in caspase-3 levels between groups, results (mean ± SEM; *n* = 4) were normalized to the loading control actin and are expressed as a percentage respect to CNT-Q7 cells. Representative immunoblots are shown. Data were analyzed by using two-way ANOVA followed by Tukey as a post-hoc test. *p < 0.05 and ***p < 0.001 as compared to CNT-Q7 cells and ###*p* < 0.001 as compared to CNT-Q111 cells.

**FIGURE 5:**
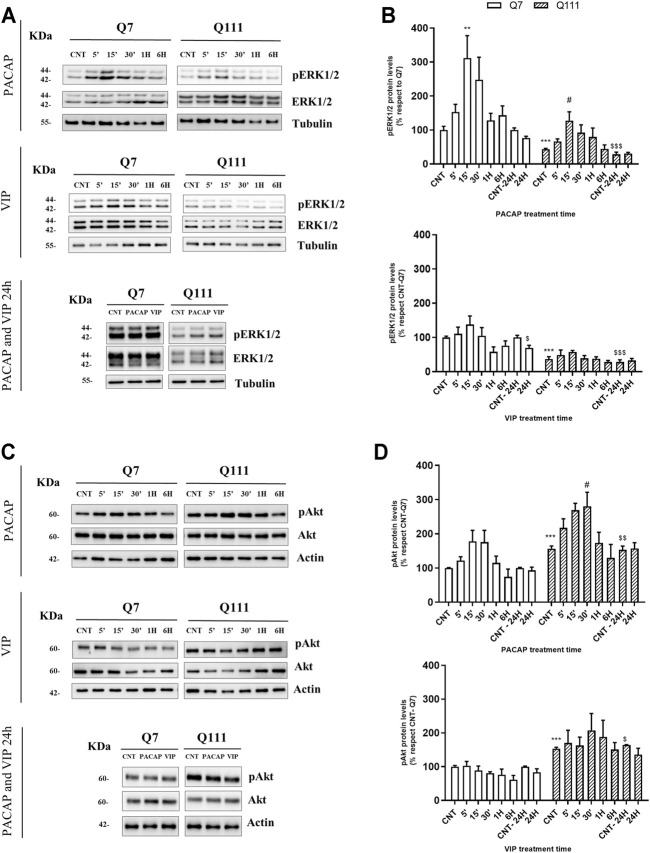
PACAP but not VIP treatment promotes the phosphorylation of ERK and Akt. STHdhQ7/Q7 (Q7) and STHdhQ111/Q111 (Q111) cells were treated with PBS (CNT), PACAP (10-7M) or VIP (10-7M) for 5, 15, 30 minutes, 1, 6 and 24 hours. Protein levels of **(A)** pERK1/2 and **(C)** pAkt (Ser473) were analyzed by Western blot. Quantification of **(B)** pERK and **(D)** pAkt was performed. As no changes were detected in ERK and Akt levels, results (mean ° SEM; n ° 4) were normalized to the loading control tubulin or actin and are expressed as a percentage respect to CNT-Q7 cells. Representative immunoblots are shown. Differences in protein basal levels between CNT-Q7 and CNT-Q111 cells were analyzed by unpaired Student’s t-test. The effect of PACAP and VIP treatments for 24 hours was analyzed by using the two-way ANOVA test followed by Tukey as post-hoc test. Treatment response over time was analyzed separately in each cell type using one-way ANOVA followed by Dunnett as *post-hoc* test. When variances between groups were not equal Welch’s correction was applied. *p < 0.05 and ***p < 0.001 as compared to CNT-Q7 cells; #*p* < 0.05 as compared to CNT-Q111 cells; $*p* < 0.05, $$*p* <0.01, and $$$*p* < 0.001 as compared to CNT-24H-Q7 cells.

**FIGURE 6:**
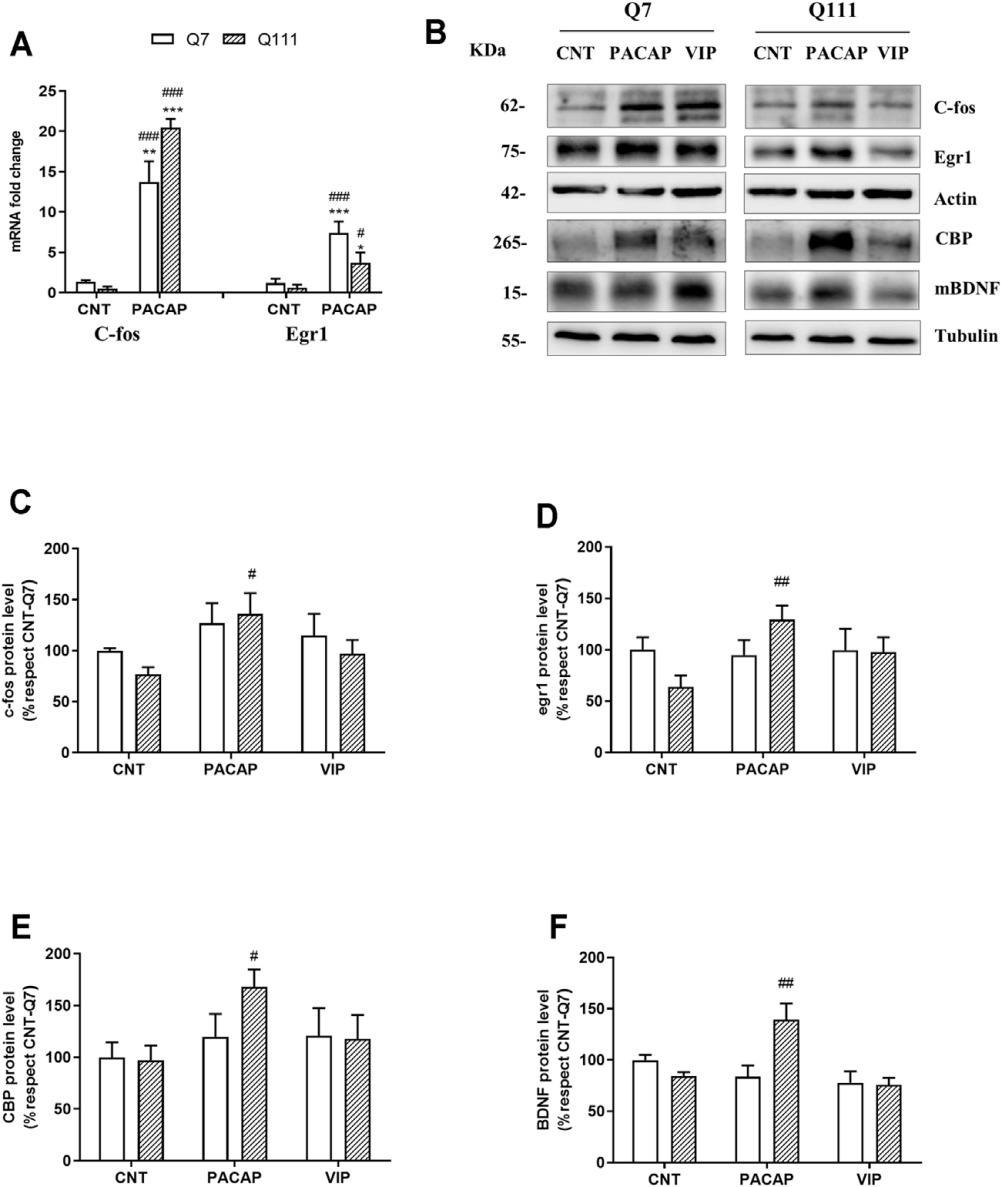
PACAP but not VIP treatment increases c-fos, egr1, CBP, and BDNF levels in STHdhQ111/Q111 cells. STHdhQ7/Q7 (Q7) and STHdhQ111/Q111 (Q111) cells were treated with PBS (CNT), PACAP (10^−7^ M) or VIP (10^−7^ M) **(A)** After 1 h of PACAP addition, mRNA levels of c-fos and egr1 were analyzed using quantitative RT-PCR. The results (mean ± SEM; *n* = 5) were normalized to GAPDH levels and are expressed as a percentage respect to CNT-Q7. **(B)** Protein levels of c-fos, egr1, and CBP were analyzed 6 h after PACAP addition and BDNF 24 h after PACAP addition. Representative immunoblots are shown. Quantification of **(C)** c-fos, **(D)** egr1, **(E)** CBP, and **(F)** mBDNF was performed. Results (mean ± SEM; *n* = 5) were normalized to the loading control tubulin or actin and are expressed as a percentage respect to CNT-Q7 cells. In all cases, data were analyzed by using two-way ANOVA followed by Tukey as post-hoc test. *p < 0.05, **p < 0.01, ***p < 0.001, as compared to CNT-Q7 cells; #*p* < 0.05; ##*p* < 0.01, and ###*p* < 0.001 as compared to CNT-Q111 cells.

**FIGURE 7:**
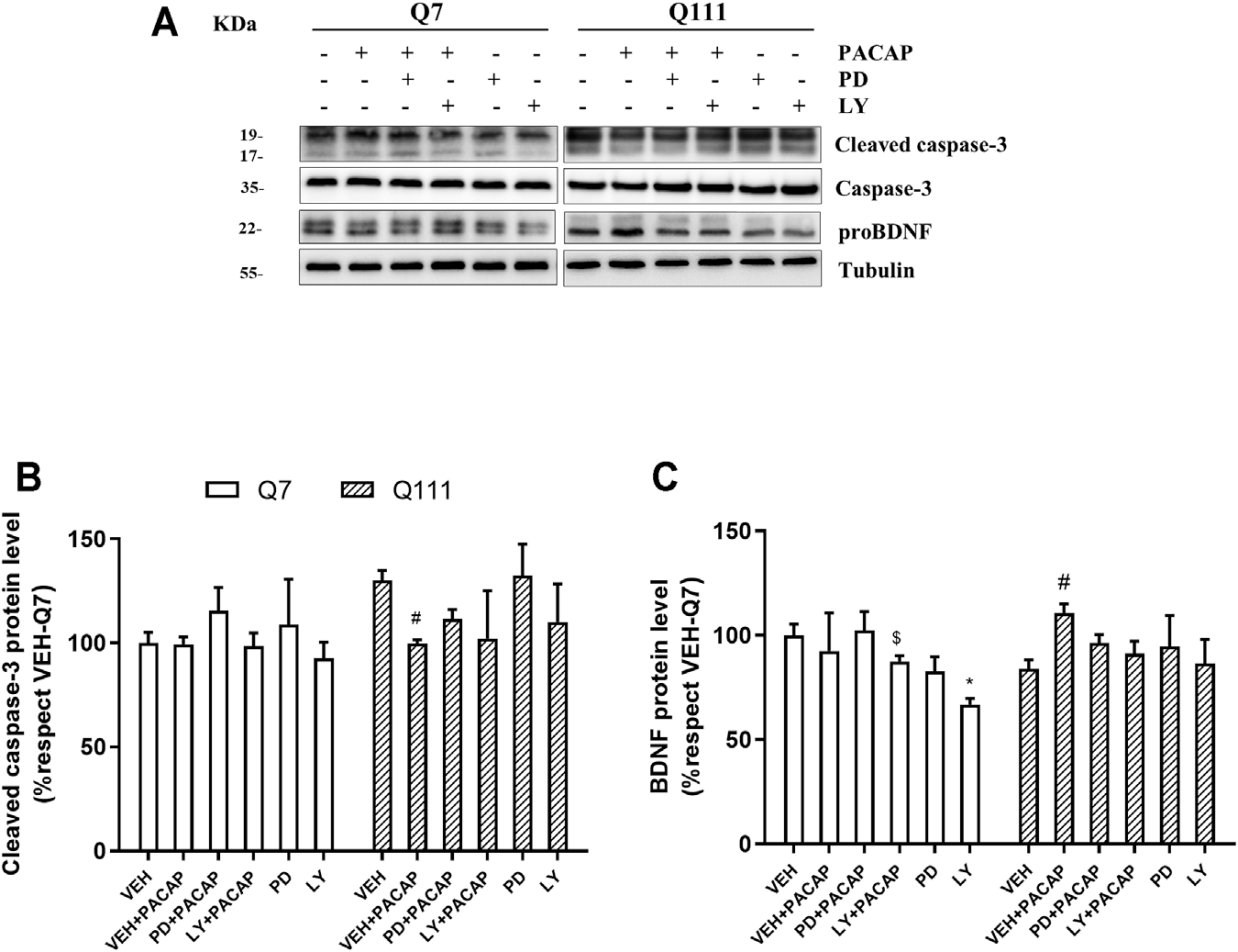
ERK and AKT pathways mediate the PACAP effect on cleaved caspase-3 and BDNF protein levels. STHdhQ7/Q7 (Q7) and STHdhQ111/Q111 (Q111) cells were treated with VEH (DMSO), PD98059 (PD, 10 µM) or LY94002 (LY, 10 µM). PACAP treatment was performed 30 min after and 24 h later, protein levels of cleaved caspase-3 and proBDNF were analyzed **(A)** Representative immunoblots are shown. Quantification of **(B)** cleaved caspase-3, and **(C)** proBDNF was performed. Results (mean ± SEM; *n* = 4) were normalized to the loading control tubulin and are expressed as a percentage respect to VEH. Treatment response was analyzed separately in each cell type and for each inhibitor. In all cases, data were analyzed by using one-way ANOVA followed by Tukey as post-hoc test. When variances between groups were not equal Welch’s correction was applied and Thamane’s T2 was used as a post hoc test. *p<0.05 as compared to VEH-Q7; #<0.05 as compared to VEH-Q111 cells; $ < 0.05 as compared to LY-Q7 cells.

**FIGURE 8:**
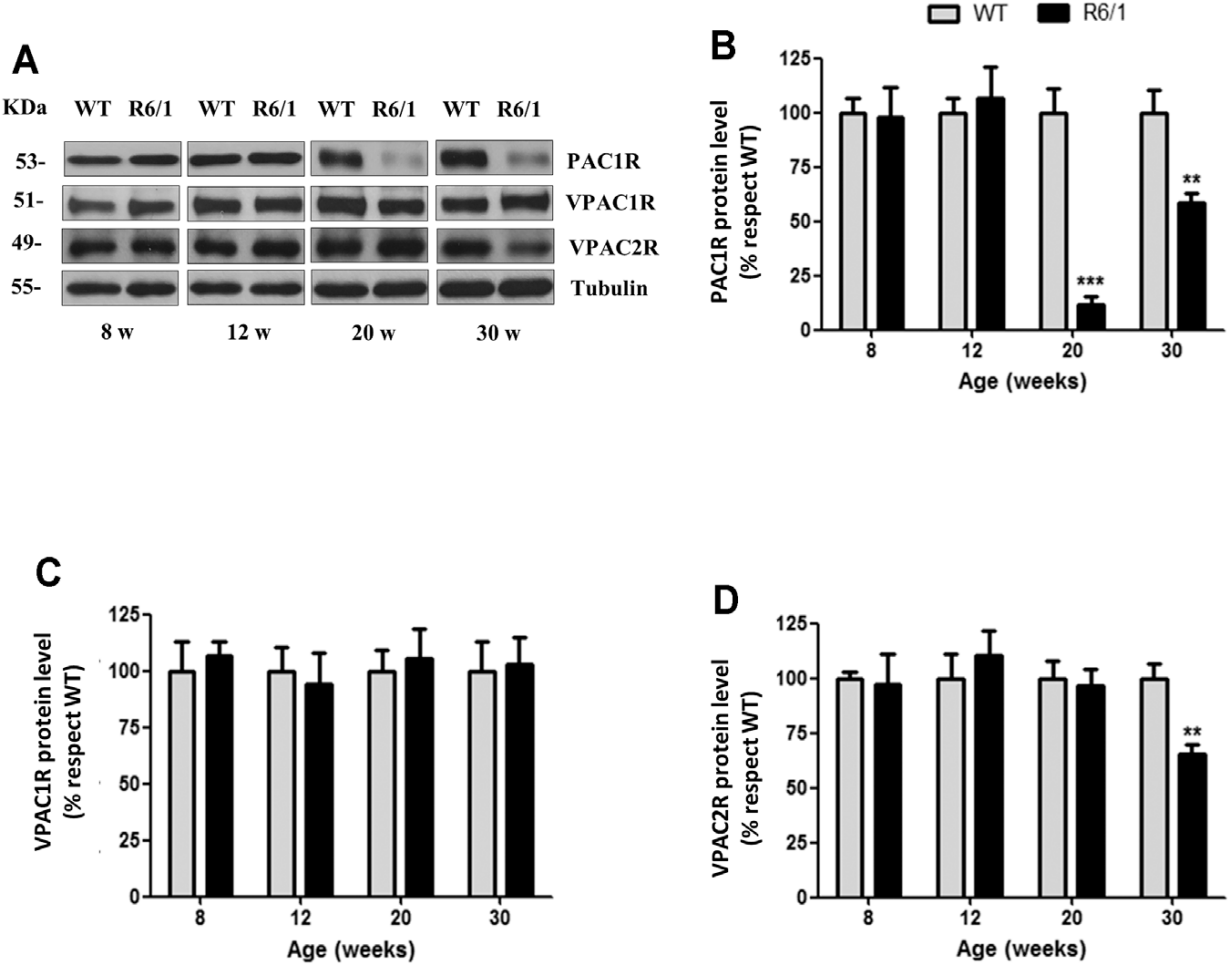
PAC1R is decreased in the striatum of R6/1 mice from week 20. Protein levels of PAC1R, VPAC1R, and VPAC2R were analyzed in striatal samples of WT and R6/1 mice at different ages by Western blot. **(A)** Representative immunoblots are shown. Quantification of **(B)** PAC1R, **(C)** VPAC1R, and **(D)** VPAC2R was performed. Results (mean ± SEM; *n* = 7) were normalized to the loading control tubulin and are expressed as a percentage respect to WT mice. Statistical analysis was performed using unpaired Student’s t-test. **p < 0.01 and ***p < 0.001 as compared to WT mice.

**FIGURE 9:**
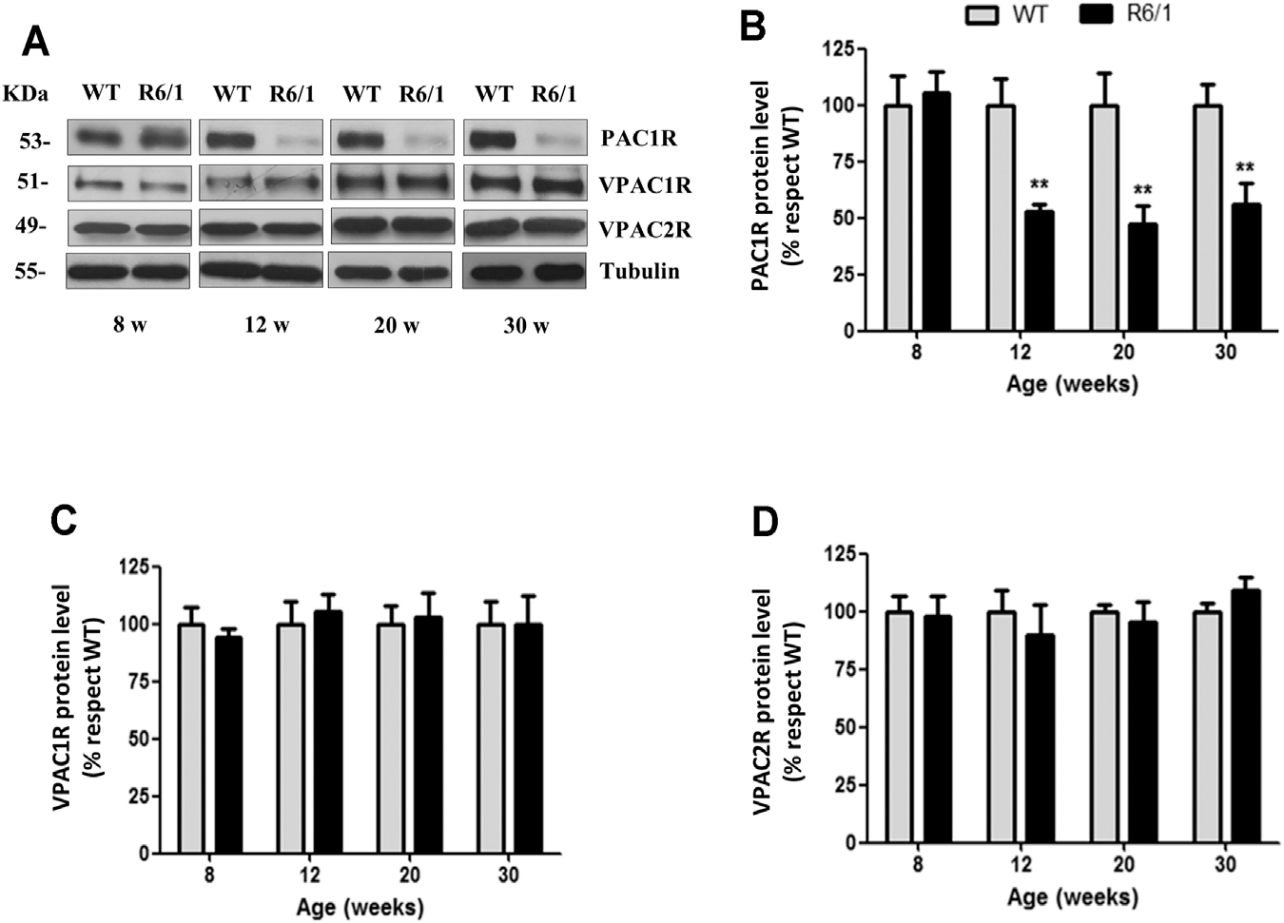
PAC1R is reduced in the motor cortex of R6/1 mice from 12 weeks of age. Protein levels of PAC1R, VPAC1R, and VPAC2R were analyzed in striatal samples of WT and R6/1 mice at different ages by Western blot. **(A)** Representative immunoblots are shown. Quantification of **(B)** PAC1R, **(C)** VPAC1R, and **(D)** VPAC2R was performed. Results (mean ± SEM; *n* = 7) were normalized to the loading control tubulin and are expressed as a percentage respect to WT mice. Statistical analysis was performed using unpaired Student’s *t*-test. **p < 0.01 as compared to WT mice.

**FIGURE 10:**
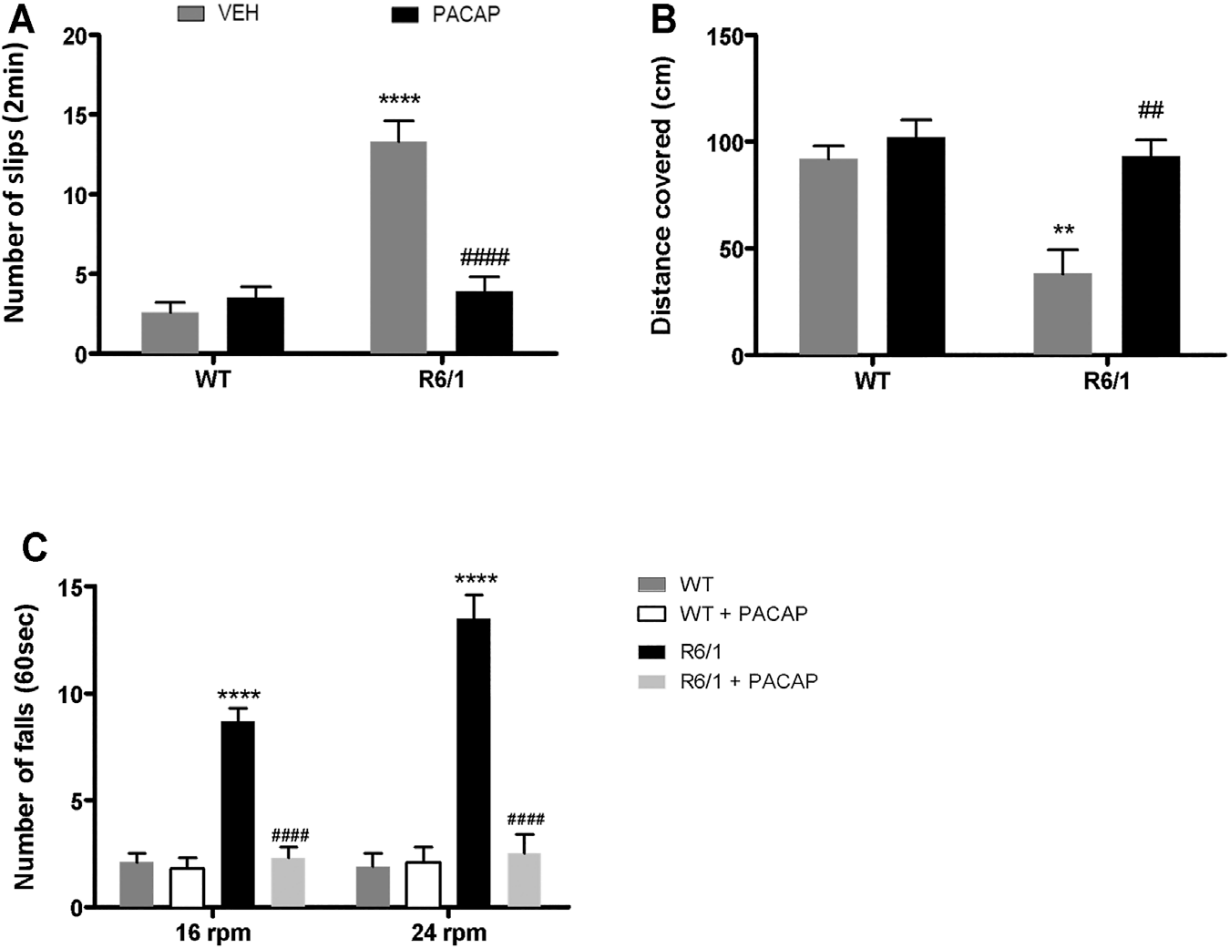
PACAP administration decreases motor deficits in R6/1 mice. At 18 weeks of age, WT and R6/1 mice were daily intranasally administrated with vehicle (VEH) or PACAP (30 μg/kg/day). The following measurements were recorded after 7 days of treatment: **(A)** number of slips and **(B)** distance covered using the balance beam task; **(C)** number of falls recorded in the rotarod test. Results are expressed as mean ± SEM (*n* = 7/group). Data were analyzed by two-way ANOVA followed by Bonferroni as post-hoc test. **p < 0.01 and ****p < 0.001 as compared to VEH-WT and ##*p* < 0.01 and ####*p* < 0.001 as compared to VEH-R6/1 mice.

**FIGURE 11:**
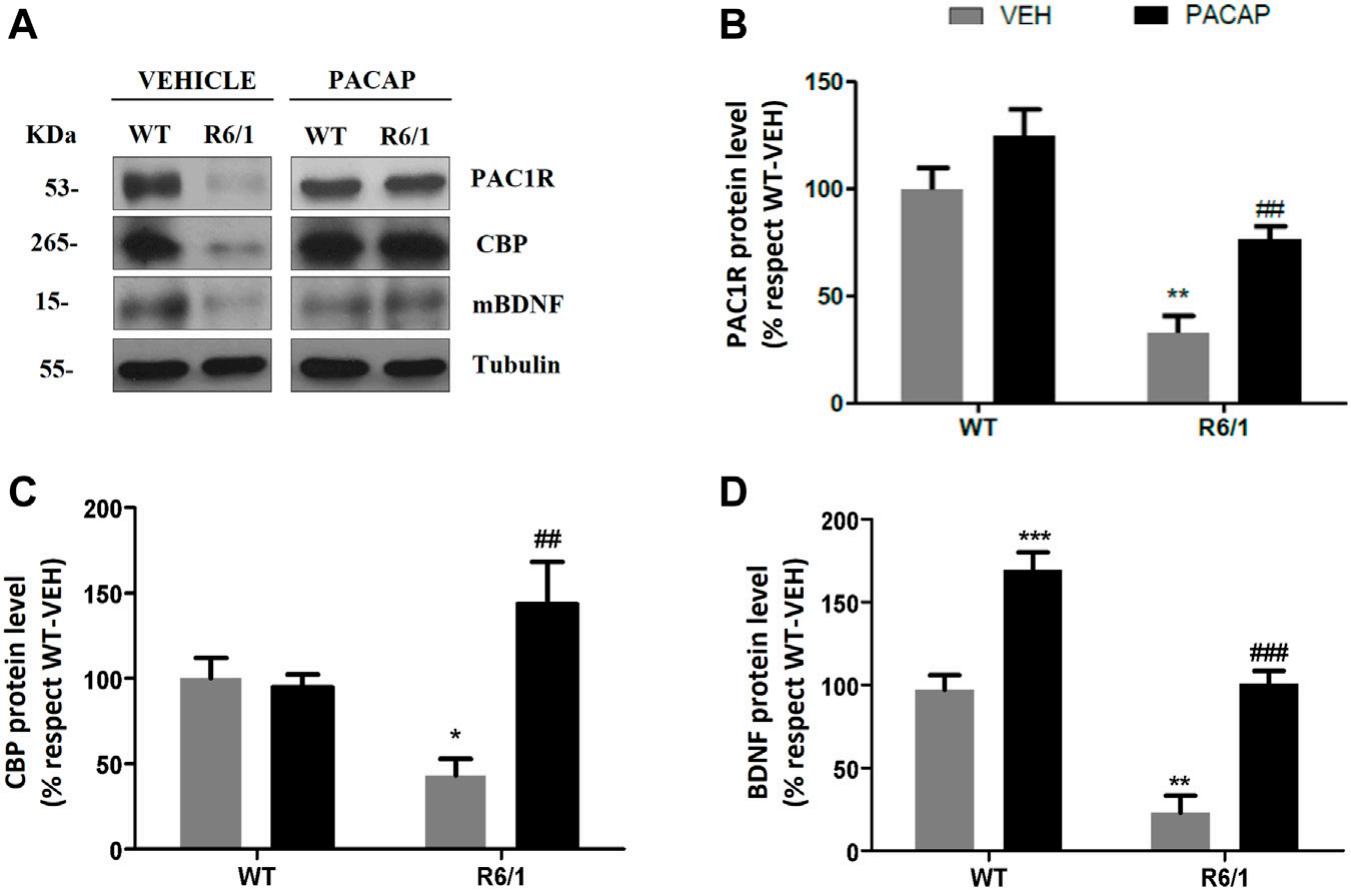
PACAP administration increases PAC1R, CBP and BDNF protein levels in the striatum of R6/1 mice. WT and R6/1 mice were daily intranasally administrated at 18 weeks of age with vehicle (VEH) or PACAP (30 μg/kg/day) for 7 days. Then, protein extracts from the striatum of all groups were subjected to Western blot for the immunodetection of PAC1R, CBP, and BDNF. **(A)** Representative immunoblots are shown. Quantification of **(B)** PAC1R, **(C)** CBP and **(D)** mBDNF was performed. Results (mean ± SEM; *n* = 7) were normalized to the loading control tubulin and are expressed as a percentage respect to VEH-WT mice. Data were analyzed by two-way ANOVA followed by Bonferroni as post-hoc test. *p < 0.05, **p < 0.01 and ***p < 0.001 as compared to VEH-WT mice and ##*p* < 0.01 and ###*p* < 0.001 as compared to VEH-R6/1 mice.

First, normal distribution was tested with Shapiro–Wilk, Kolmogorov–Smirnov, and d’Agostino-Pearson omibus normality tests. If one of these tests was passed, statistical analysis was completed using parametric statistical analysis. Levene’s method for variances was used to determine equal or unequal variance. In experiments with normal distribution, we used unpaired two-sided Student’s test to compare two groups (Figures 3, 5, 8, 9). For multiple comparisons, we used one-way ANOVA with Dunnett or Tukey’s as post hoc tests when we study how treatments affects a response variable (Figures 5, 7) and two-way ANOVA with the Tukey’s or the Bonferroni’s post hoc test to determine how genotype and treatments affect a response variable (Figures 2–6, 10, 11). When variances were unequal, Welch’s *t*-test for comparing two groups and Welch’s ANOVA test with Dunnett or Thamane’s T2 as a post hoc tests for comparing multiple groups were applied. All data are expressed as mean ± SEM and values of *p* < 0.05 were considered statistically significant. Grubbs’ test was performed to determine the significant outlier values. The statistical analysis was performed using GraphPad Prism (Version 9.0.0, GraphPad Software, San Diego, California United States).

## Results

### PACAP Protects From Mutant Htt-Mediated Cell Death

We studied the protective capacity of PACAP against mHtt in immortalized striatal cell lines expressing WT (STHdhQ7/Q7) or mutant (STHdhQ111/Q111) Htt. The treatment of PACAP (10^–7^ M) for 24 h resulted in a significant increase in cell survival in STHdhQ111/Q111 cells [Two-way ANOVA, Interaction effect: *F*_(1,12)_ = 72.90, *p* < 0.0001, genotype effect: *F*_(1,12)_ = 240, *p* < 0.0001, PACAP treatment effect: *F*_(1,12)_ = 94.64, *p* < 0.0001] (Figure 2A) as measured by the MTT assay. Additionally, the treatment of PACAP reduced the number of apoptotic nuclei [Two-way ANOVA, Interaction effect: *F*_(1,12)_ = 58.26, *p* < 0.0001, genotype effect: *F*_(1,12)_ = 115.5, *p* < 0.0001, PACAP treatment effect: *F*_(1,12)_ = 128.3, *p* < 0.0001] (Figures 2B,C). In STHdhQ7/Q7 cells, although no effect was observed on cell survival, PACAP treatment had the capacity to reduce the number of basal apoptotic nuclei (Figure 2).

### PACAP Effect is Associated to the Increase of PAC1R in STHdhQ111/Q111 Cells

We analyzed protein levels of PACAP receptors in STHdh cells (Figure 3A). We found STHdhQ111/Q111 present diminished protein levels of PAC1R (Student’s *t*-test: *t* = 6.257, *p* = 0.0002) and VPAC1R (Student’s *t*-test: *t* = 2.574, *p* = 0.0329) respect to STHdhQ7/Q7 cells (Figure 3B). Related to VPAC2R protein levels, we did not find significative differences between cell lines (Student’s *t*-test: *t* = 1.160, *p* = 0.2793) (Figure 3B). As the specific receptor of PACAP is PAC1R, we studied mRNA and protein levels of PAC1R in PACAP-treated STHdh cells. STHdhQ111/Q111 cells displayed reduced PAC1R mRNA and the addition of PACAP for 24 h resulted in a raise of PAC1R mRNA in both STHdhQ7/Q7 and STHdhQ111/Q111 cells (Figure 3C) but this increase was not enough to rise PAC1R protein levels (Figure 3D).

### PACAP Reduced Cleaved Caspase-3 Levels in STHdhQ111/Q111 Cells

We also explored the ability of PACAP to reduce the levels of cleaved caspase-3 induced by the presence of mHtt (Figure 4). We observed STHdhQ111/Q111 presented higher cleaved caspase-3 protein levels than STHdhQ7/Q7 and that the addition of PACAP resulted in a decrease of this protein in STHdhQ111/Q111 cells [Two-way ANOVA, Interaction effect: *F*_(1,11)_ = 4.91, *p* < 0.0487, genotype effect: *F*_(1,11)_ = 29.6, *p* < 0.0002, PACAP treatment effect: *F*_(1,11)_ = 36.1, *p* < 0.0001], confirming the antiapoptotic effect of PACAP. To know the role of PAC1R, we also studied the effect of VIP, a peptide with high affinity for VPAC1R and VPAC2R and low affinity for PAC1R. The treatment with VIP also reduced the cleaved caspase-3 levels [Two-way ANOVA, Interaction effect: *F*_(1,11)_ = 6.84, *p* = 0.0240, genotype effect: *F*_(1,11)_ = 11.4, *p* = 0.0061, VIP treatment effect: *F*_(1,11)_ = 63.9 *p* < 0.0001] (Figure 4) indicating that the stimulation of all PACAP receptors have the capacity to reduce cleaved caspase-3 levels. As caspase-3 works by activation of pro-caspase-3, we did not perform the mRNA quantification of this protein.

### PACAP Promoted the Activation of Pro-Survival and Neurotrophic Proteins in STHdhQ111/Q111 Cells by the Specific Activation of PAC1R

We next analyzed by Western blot the levels of phospho-ERK (pERK) and phospho-Akt (pAkt), two pro-survival pathways altered in HD. As already described (Gines et al., 2003a), STHdhQ111/Q111 cells presented reduced pERK (Student’s t-test = 9.410, *p* < 0.0001) (Figures 5A,B) and increased pAkt **(**Welch’s *t*-test: *t* = 11.90 *p* < 0.0001) (Figures 5C,D) basal levels compared to STHdhQ7/Q7 cells. PACAP induced a rapid and transient increase in pERK [One-way ANOVA: *F*_(5,18)_ = 2.876, *p* = 0.0443] (Figures 5A,B) and pAkt [One-way ANOVA: *F*_(5,18)_ = 4.275, *p* = 0.0097] (Figures 5C, D) in STHdhQ111/Q111 cells, after 15 and 30 min of treatment respectively. This activation is associated to PAC1R, as no stimulation of neither pERK nor pAkt is observed in STHdhQ111/Q111 cells treated with VIP (Figure 5). As ERK and Akt activation is mediated through a phosphorylation process, we did not perform the mRNA quantification of these proteins. Then, we studied the protein levels of c-fos and egr1, two immediate early genes related to neuronal survival. The treatment with PACAP enhanced the levels of both c-fos and egr1 at 1 and 6 h (Figures 6A–D) in STHdhQ111/Q111 cells [Two-way ANOVA, PACAP treatment effect: *F*_(1,16)_ = 8.778 *p* = 0.0092; Two-way ANOVA, Interaction effect: *F*_(1,15)_ = 7.54, *p* = 0.0150, PACAP treatment effect: *F*_(1,15)_ = 5.55, *p* = 0.0325 respectively]. We also found that the addition of PACAP to STHdhQ111/Q111 cells promoted a significant increase in CBP [PACAP treatment effect: *F*_(1,16)_ = 7.01, *p* < 0.0176] (Figure 6E), the mature form BDNF [Two-way ANOVA, Interaction effect: *F*_(1,16)_ = 12.19, *p* = 0.0030] (Figure 6F) and the proform of BDNF (proBDNF) [Two-way ANOVA, PACAP treatment effect: *F*_(1,16)_ = 12.18, *p* = 0.0030] (data not shown) protein levels, important proteins related to neuroprotective and neurotrophic activity. The enhanced expression of these proteins is associated to PAC1R activation, as no changes were observed in c-fos (Figure 6C), egr1 (Figure 6D), CBP (Figure 6E), and BDNF (Figure 6F) expression when we treated STHdhQ111/Q111 cells with VIP.

### ERK and Akt Pathways Mediate the Reduction of Cleaved Caspase-3 and the Increase of BDNF Promoted by PACAP

To know whether activation of pERK and pAkt by PACAP in STHdhQ111/Q111 cells is involved to the antiapoptotic and neurotrophic effect, we added pharmacological inhibitors of ERK and Akt, PD98059 (PD) and LY294002 (LY) respectively, 30 min before PACAP treatment, and protein levels of cleaved caspase-3 and BDNF were measured 24 h later (Figure 7A). The presence of PD and LY blocked the reduction of cleaved caspase-3 [PD: Welch’s ANOVA: *W*_(3,5.705)_ = 10.88, *p* = 0.0088; LY: Welch’s ANOVA: *W*_(3,5.379)_ = 8.958, *p* = 0.0159] (Figure 7B) and the increase of proBDNF [PD: Welch’s ANOVA: *W*_(3, 6.466)_ = 5.361, *p* = 0.0351; LY: Welch’s ANOVA: *W*_(3,6.456)_ = 5.734, *p* = 0.0303] (Figure 7C) mediated by PACAP in STHdhQ111/Q111 cells. In STHdhQ7/Q7 cells no variation was detected in cleaved caspase-3 levels after different treatments (Figure 7B). However, the presence of LY reduced proBDNF levels, which increased significantly after PACAP addition [Welch’s ANOVA: *W*_(3, 5.854)_ = 11.37, *p* = 0.0074] (Figure 7C).

### PAC1R Protein Levels Are Reduced in the Striatum and Motor Cortex of R6/1 Mice

To know the role of PACAP receptors in the HD motor dysfunction, the protein levels of PAC1R, VPAC1R, and VPAC2R was investigated in striatal samples from WT and R6/1 mice at different stages of the disease by Western blot (Figure 8A). We observed a strong decrease in PAC1R at 20 weeks of age, when the motor discoordination is present [Student’s *t*-test: *t* = 19.53, *p* < 0.0001] (Figure 8B). This reduction was still detected at 30 weeks of age [Student’s *t*-test: *t* = 9.742, *p* < 0.0001], but to a lower level. Regarding VPAC1R and VPAC2R, no differences were detected between WT and R6/1 mice, with exception of VPAC2R which was reduced at 30 weeks of age in R6/1 mice [Student’s *t*-test: *t* = 11.87, *p* < 0.0001] (Figures 8C,D). The analysis of motor cortex from WT and R6/1 mice by Western blot (Figure 9A) revealed a reduction of PAC1R from 12 weeks of age [12w: Student’s *t*-test: *t* = 9.840, *p* < 0.0001; 20w: Student’s *t*-test: *t* = 8.141, *p* < 0.0001; 30w: Student’s *t*-test: *t* = 8.424, *p* < 0.0001] (Figure 9B). In contrast, no differences in VPAC1R and VPAC2R protein levels were observed, at any age analyzed (Figures 9C,D).

### PACAP Administration Improves Motor Deficits of R6/1 Mice

To determine whether PACAP treatment could result in motor function improvement, daily intranasal administration of PACAP (30 μg/kg/day) was performed for one week to WT and R6/1 mice at 18 weeks of age. Then, motor performance of these animals were analyzed using the balance beam and rotarod tests. In the balance beam test, PACAP treatment reduced the number of slips (Figure 10A) and increased the distance covered (Figure 10B) by R6/1 mice compared to vehicle-injected congeners [nº of slips: Two-way ANOVA, Interaction effect: *F*_(1,24)_ = 204.6, *p* < 0.0001, genotype effect: *F*_(1,24)_ = 240.2, *p* < 0.0001, PACAP treatment effect: *F*_(1,24)_ = 142.1, *p* < 0.0001; distance covered: Two-way ANOVA, Interaction effect: *F*_(1,24)_ = 48.91, *p* < 0.0001, genotype effect: *F*_(1,24)_ = 96.38, *p* < 0.0001, PACAP treatment effect: *F*_(1,24)_ = 99.87, *p* < 0.0001]. In the rotarod test, administration of PACAP promoted a strong and significant reduction in the number of falls at both 16 [Two-way ANOVA, Interaction effect: *F*_(1,24)_ = 154.1, *p* < 0.0001, genotype effect: *F*_(1,24)_ = 216.8, *p* < 0.0001, PACAP treatment effect: *F*_(1,24)_ = 184.1, *p* < 0.0001] and 24 rpm [Two-way ANOVA, Interaction effect: *F*_(1,24)_ = 286.6, *p* < 0.0001, genotype effect: *F*_(1,24)_ = 315.9, *p* < 0.0001, PACAP treatment effect: *F*_(1,24)_ = 258.6, *p* < 0.0001] (Figure 10C). In WT mice, PACAP treatment had no effect on motor performance (Figure 10).

### Amelioration of Motor Performances in PACAP-Treated R6/1 Mice is Associated With a Recovery of PAC1R, CBP, and BDNF Protein Levels in the Striatum

We next analyzed protein levels of PAC1R in the striatum of WT and R6/1 mice after PACAP treatment (Figure 11A). We observed that the administration of PACAP in R6/1 mice significantly increased PAC1R protein levels when compared to vehicle-treated R6/1 mice [Two-way ANOVA, Interaction effect: *F*_(1,24)_ = 6.559, *p =* 0.0171, genotype effect: *F*_(1,24)_ = 236.7, *p* < 0.0001, PACAP treatment effect: *F*_(1,24)_ = 79.36, *p* < 0.0001] (Figure 11B). No changes were observed in PACAP-treated WT mice. Given the importance of CBP and BDNF striatal loss in the pathophysiology of HD, we next explored their protein levels in the striatum of PACAP-treated R6/1 mice (Figure 11A). Results show that striatum of R6/1 mice at 19 weeks of age displayed a strong reduction in CBP (Figure 11C) and BDNF (Figure 11D) protein levels compared to WT animals. But interestingly, this decrease in CBP and BDNF observed in vehicle-treated R6/1 mice was totally blocked by PACAP treatment (Figures 11C,D). In WT mice, PACAP also enhanced BDNF protein levels (Figure 11D) although it did not induce any changes in CBP protein expression (Figure 11C) [CBP: Two-way ANOVA, Interaction effect: *F*_(1,24)_ = 90.21, *p <* 0.0001, genotype effect: *F*_(1,24)_ = 0.7043, *p* = 0.0496, PACAP treatment effect: *F*_(1,24)_ = 75.82, *p* < 0.0001; BDNF: Two-way ANOVA, Interaction effect: *F*_(1,24)_ = 1.478, *p =* 0.2360, genotype effect: *F*_(1,24)_ = 386, *p* < 0.0001, PACAP treatment effect: *F*_(1,24)_ = 384.1, *p* < 0.0001].

## Discussion

The loss of striatal neurons is a key feature for the apparition and progression of HD motor discoordination (Guo et al., 2012). Here, we prove using an *in vitro* HD model, the STHdhQ111/Q111 cell line, that the presence of PACAP promotes the survival of striatal cells expressing mHtt from apoptotic cell death by reducing cleaved caspase-3 levels. Additionally, we show that an intranasal administration of PACAP at the onset of motor symptoms in R6/1 mice ameliorates motor discoordination and balance deficits. An antiapoptotic effect of PACAP involving an inhibition of caspase-3 activity has also been shown in cellular models of AD (Onoue et al., 2002) and PD (Wang et al., 2005) with Aβ- and rotenone-induced toxicity, respectively. However, the capacity of PACAP to rescue motor symptoms in neurodegenerative disorders has been poorly explored. Years ago, PACAP was found to attenuate behavioral deficits in an excitotoxic HD model by protecting neurons from a quinolinic-induced unilateral lesion of the striatum (Tamás et al., 2006). Similarly, PACAP also was able to prevent motor and behavioral deficits in two different murine models of PD induced by prostaglandin J2 (Shivers et al., 2014) and 6-OHDA (Reglodi et al., 2004), respectively. Therefore, our work is the first study, at our knowledge, showing that PACAP induces a motor recovery in a transgenic mouse model of neurodegenerative disorders. In addition, it has been published the ability of PACAP to improve cognitive function in transgenic AD (Rat et al., 2011) and we previously observed that PACAP promotes a recovery of cognitive function in HD (Cabezas-Llobet et al., 2018). Moreover, our study supports that intranasal administration of PACAP is effective, as other authors have seen before (Polanco et al., 2016; Cherait et al., 2021; Guo et al., 2021). Altogether, it is tentative to propose PACAP as a promising therapeutic tool for HD and intranasal administration could be considered as therapeutical method for possible future human use.

In the pathology of HD, loss of BDNF has been strongly associated with the degeneration of the striatum and development of motor discoordination (Gavaldà et al., 2004; Giralt et al., 2009; Giralt et al., 2010). Reduction of BDNF levels has been found in HD patients (Ferrer et al., 2000) as well as in several animal models contributing to the onset and severity of the symptoms (Canals et al., 2004; Pang et al., 2006; Zuccato and Cattaneo, 2007). Importantly, BDNF expression is mediated by CREB and CBP (Esvald et al., 2020), and CBP is strongly downregulated in HD (Nucifora et al., 2001; Jiang et al., 2006; Giralt et al., 2012). Here, we observed that PACAP enhances BDNF expression in STHdhQ111/Q111 cells and in the striatum of PACAP-treated R6/1 mice, indicating that it can induce neurotrophic activity. This effect was accompanied by an increase in CBP protein levels, both in STHdhQ111/Q111 cells and the striatum of R6/1 mice treated with PACAP. In the context of cognition, PACAP beneficial effects have been related to an increase of BDNF expression in AD and HD murine models (Rat et al., 2011; Cabezas-Llobet et al., 2018; Solés-Tarrés et al., 2020). Additionally, BDNF has been proposed as the mediator of PACAP actions in the context of learning and memory (Ladjimi et al., 2019) and in PACAP neuroprotective effect observed in cortical neurons (Frechilla et al., 2001). The capacity of PACAP to activate CBP has been described in activated microglia where PACAP increases CBP-binding to CREB (Delgado, 2002) and some authors point out that PACAP neuroprotective effect is CREB-dependent (Baxter et al., 2011; Emery and Eiden, 2012; Brown et al., 2013). In AD, the restoration of BDNF levels by the modulation of CBP is a therapeutic strategy that has proven to be effective (Caccamo et al., 2010). Thus, our findings suggest that PACAP protects striatal cells and improves motor deficits in R6/1 mice by raising CBP protein levels and therefore by facilitating the expression and release of BDNF in the striatum.

The reduction of immediate early genes (iEGs) has been observed in the striatum of human samples and in HD mouse models (Desplats et al., 2006) and it has been proposed as a key mechanism leading to striatal degeneration in HD (Roze et al., 2011). We found that in STHdhQ111/Q111 cells the protective effect of PACAP is associated with the increase in c-fos and egr1 expression, two iEGs. Both c-fos and egr1 are proteins involved in neuronal activity, plasticity and survival (Zhang et al., 2002; West and Greenberg, 2011; Keilani et al., 2012). Noteworthy, PACAP has been previously described to stimulate c-fos and egr1 expression in neurons (Vaudry et al., 1998; Ravni et al., 2008). Moreover, these genes are reduced in the striatum of R6/1 mouse model at ages where motor symptoms are expressed (Anglada-Huguet et al., 2016). In HD, this reduction could be due to the abnormal decrease in CBP protein levels, which has been reported to cause a downregulation of CREB dependent genes (Nucifora et al., 2001; Jiang et al., 2006; Giralt et al., 2012), such as BDNF, but also c-fos and egr1 (Shieh et al., 1998; Tao et al., 1998; Lonze and Ginty, 2002). Therefore, we propose that PACAP promotes the expression of c-fos and egr1 by increasing CBP. However, the effect of PACAP does not seem to be restricted to c-fos and egr1 iEGs, as in various neuronal models, PACAP promotes transcriptional changes of other genes (Frechilla et al., 2001; Falluel-Morel et al., 2004; Aubert et al., 2006; Ravni et al., 2006; Dejda et al., 2008). Altogether, our results show that PACAP induces the survival of cells expressing mHtt and promotes the increase in CBP and BDNF, but also modulates the expression of iEGs in the striatum to improve the cellular functions.

PACAP receptors are G protein-coupled receptors that can activate multiple signaling cascades including ERK1/2 and Akt (Dickson and Finlayson, 2009; Vaudry et al., 2009), the two main pathways regulating cell survival and neurotrophic effects (Rai et al., 2019). An altered expression and activity of ERK1/2 and Akt have been found in multiple preclinical HD models and human tissue samples from people who died from HD, showing that it contributes to the development of the disease (Humbert et al., 2002; Colin et al., 2005; Apostol et al., 2006; Roze et al., 2008). Accordingly, we found in STHdhQ111/Q111 diminished basal ERK1/2 activation and elevated basal Akt activation as also reported by other authors before (Gines et al., 2003a; Ginés et al., 2010). The activation of these pathways has been demonstrated to be protective against mHtt-toxicity (Humbert et al., 2002; Maher et al., 2011; Sarantos et al., 2012; Geva et al., 2016). In accordance, the present study establishes that PACAP activates both ERK1/2 and Akt inhibitions in striatal cells expressing mHtt and that this activation mediates the neuroprotective and neurotrophic effects of PACAP.

PACAP acts through three receptors, i.e. PAC1R, VPAC1R and VPAC2R (Vaudry et al., 2009; Harmar et al., 2012). In the striatum of R6/1 mice, PAC1R is strongly downregulated from the onset of motor dysfunction. Interestingly, a specific reduction of PAC1R is also found in the motor cortex of R6/1 and a decrease of PAC1R expression is established in STHdhQ111/Q111 cells. This is in line with the previously reported reduction levels of PAC1R in the hippocampus of R6/1 mice and HD patients (Cabezas-Llobet et al., 2018). It has been found in other neurodegenerative diseases a decrease of PAC1R protein levels in brain areas affected by the neuropathology. In PD it has been described a downregulation of PAC1R in the caudate and putamen brain regions of 1-methyl-4-phenyl-1,2,3,6-tetrahydropyridine (MPTP) treated macaque animals (Feher et al., 2018) and similar results have been obtained in motor cortex of amyotrophic lateral sclerosis (ALS) patients (Bonaventura et al., 2018). Therefore, it is tentative to suggest that the age-related decrease of PAC1R expression observed in R6/1 mice, could be an event that occurs in HD brain playing a role in the development and progression of HD symptoms. The activation of VPAC receptors has been associated to the increased activity of pathways involved in neuroprotective and neurotrophic actions, such as ERK1/2 (Vaudry et al., 2000; Langer, 2012). We observed a reduction of VPAC1R in STHdhQ111/Q111 cells and a protective effect of VIP, indicating a posible beneficial role of VPAC receptors. However, the PAC1R signaling seems crucial to activate protective and neurotrophic mechanisms. In addition, we detected reduced VPAC2R in the striatum of R6/1 mice at 30 weeks of age indicating a possible role of this receptor at later phases. The role of VPACR is not restricted to neurons since in astrocytes, VPAC receptors are related to the release of neuroprotective and neurotrophic factors (Brenneman et al., 1999). Importantly, it has been suggested that VPAC2R plays a major role than VPAC1R in the astrocytic-mediated neuroprotection and neurotrophic activity (Zusev and Gozes, 2004; Passemard et al., 2011). Altogether, we suggest that the reduced PACAP-mediated signaling in the striatum is mainly depending of PAC1R but that it could be aggravated by the decrease of VPACR.

We found that motor improvement in PACAP-treated R6/1 mice was linked to the increase of PAC1R levels in striatum. Interestingly, this is not restricted to the HD striatum as we previously reported that PACAP-mediated improvement of cognitive function in R6/1 mice was associated with hippocampal PAC1R upregulation (Cabezas-Llobet et al., 2018). These results are in line with other studies in which PACAP has shown to have the capacity to trigger PAC1R expression (Hashimoto et al., 2000; Rat et al., 2011). Remarkably, the recovery of PAC1R in striatum of R6/1 mice did not equal PAC1R levels of WT mice although the behavioral performance of these animals was back to normal. These outcomes suggest that PACAP does not only stimulates PAC1R expression but also potentiates the activity of available PAC1R, enhancing its associated pro-survival and neurotrophic signaling cascade. In accordance, the BDNF and CBP protein levels were completely rescued in the striatum of PACAP-treated R6/1 mice. Results obtained in STHdhQ111/Q111 striatal cell line support this idea, as the addition of PACAP to these cells results in neuroprotection without increasing PAC1R protein levels. Thus, we suggest PACAP beneficial effects in HD may occur through both the upregulation and stimulation of PAC1R in striatum. However, because PAC1R is expressed thorough the brain, and intranasal administration does not a specific effect in striatum, PACAP action in other brain areas could occurred to improve the behavioral performance of R6/1 mice. Importantly, PAC1R is highly expressed in the cerebral cortex (Solés-Tarrés et al., 2020), the main input region of the striatum. As an important reduction of PAC1R protein levels is detected in the motor cortex and as dysregulation of corticostriatal pathway is a main event in the HD motor dysfunction (Bunner and Rebec, 2016), it is tentative to suggest that PACAP administration also promotes an effect on motor cortex, and therefore, the corticostriatal pathway. Hence, further studies are needed to investigate PACAP effect and PAC1R in other brain areas, especially in motor cortex, affected in HD.

Although neuroprotective activities of PACAP have usually been related to the activation of PAC1R, some *in vivo* studies demonstrate that activation of VPAC receptors can be also neuroprotective (Tunçel et al., 2012; Korkmaz et al., 2019). To uncover the contribution of PAC1R and VPAC1R/VPAC2R in STHdhQ111/Q111 cell line, the effect of PACAP was compared to the effect of VIP, a peptide with equal affinity for VPAC1R and VPAC2R and less affinity for PAC1R. Results show that VIP treatment inhibits caspase-3 activation, but does not enhance the expression and activation of ERK, Akt, c-fos, egr1, and BDNF as PACAP treatment do, which suggests that although activation of VPAC1R/VPAC2R induces protection against mHtt, the activation of PAC1R is required to activate pro-survival and neurotrophic mechanisms contributing to the long lasting functionality of striatal cells in HD models. Similar results have been obtained in other neuronal models where VIP prevents brain atrophy in 5xFAD AD mice (Korkmaz et al., 2019) and protects striatum from 6-OHDA in a rat model of PD through its antioxidant and anti-apoptotic actions (Tunçel et al., 2012). However, several studies showed that PACAP treatment but not VIP rescues the adverse effect of MPP+ in SH-SY5Y cells (Doan et al., 2011) or prevents cerebellar granule cells from C2-ceramide-induced apoptosis (Vaudry et al., 2003).

It is important to point out some limitations of the study. Although pharmacological inhibitors of ERK and Akt are widely used, they are not exclusively selective for these proteins. In addition, in some experiments a cell line was used, which has lower biological relevance than primary cultures. However, in the present study, we used a well-established HD striatal cell line that it has been extensively used to study the pathogenic mechanisms of mutant huntingtin, such as post-Golgi trafficking (Del Toro et al., 2006; Del Toro et al., 2009) or transcription factors (Xifró et al., 2011; Anglada-Huguet et al., 2012). It should also be kept in mind that we observed some differences between the striatal cell line and the dissected striatal tissue. These differences could be due by the presence or absence of glial cells. This will be an interesting point to investigate since most neuroprotective actions endorsed by VPAC receptors in neurodegenerative diseases involve glial cells (Solés-Tarrés et al., 2020). In contrast, we also detect similar results between both models, such as the reduction of PAC1R and the PACAP-mediated increase of CBP and BDNF. These similarities and the fact that VPAC2R activation has been associated to the side effects of PACAP through its periphery distribution (Fizanne et al., 2004), support the view that PAC1R activation seems to be the most approachable therapy in the field of neurodegenerative diseases. Related to this, it has been demonstrated recently that a synthetic analogue of PACAP with preference to PAC1R and relatively low affinity towards the VPAC receptors is as efficient as PACAP protecting against MPTP toxicity in a PD animal model (Lamine et al., 2016).

**FIGURE 12:**
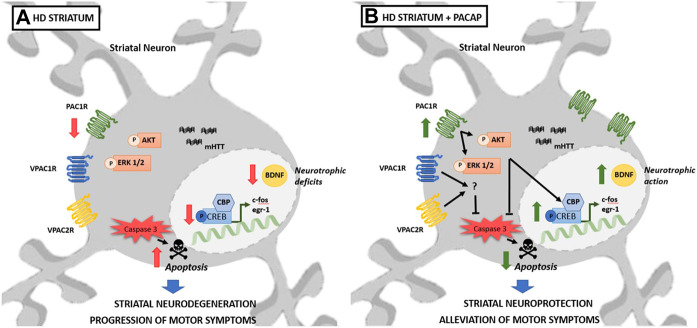
Proposed mechanisms by which PACAP protects striatal neurons from mHtt toxicity and performs motor function. **(A)** HD striatum: striatal neurons expressing mHtt exhibit diminished protein levels of PAC1R from the onset of motor symptoms, without changes in VPAC1R and VPAC2R. The decrease of PAC1R is associated with the reduction of early genes and CBP and with the downregulation of BDNF conducing to cell death and neurotrophic deficits leading to motor discoordination. **(B)** HD striatum + PACAP: PACAP administration protects striatal cells and enhances motor function. These effects are associated to the increase in PAC1R, to the phosphorylation of ERK and Akt and to the enhanced expression of early genes, CBP and BDNF. Although activation of VPAC1R and VPAC2R protects striatal cells from mHtt-induced toxicity, the activation of PAC1R is crucial to induce the antiapoptotic and neurotrophic effect of PACAP through the activation of described signaling.

In conclusion, with the present study we demonstrate for the first time that administration of PACAP protects striatal cells from toxicity induced by mHtt and improves the motor function in R6/1 mice. These beneficial effects are mediated by the activation of PAC1R in the striatum, resulting in a stimulation of pro-survival pathways such as ERK1/2 and Akt, leading to antiapoptotic and neurotrophic effects (Figure 12). Although the activation of VPAC1R/VPAC2R could have a beneficial effect, the correlation between the onset of motor discoordination and a specific decrease in PAC1R (Figure 12) strongly suggest that activation of PAC1R is a key therapeutic target for the treatment of HD.

## Data Availability

The original contributions presented in the study are included in the article/Supplementary Material, further inquiries can be directed to the corresponding author.
