# Supplementary material for: Pituitary Adenylate Cyclase-Activating Polypeptide (PACAP) Protects Striatal Cells and Improves Motor Function in Huntington’s Disease Models: Role of PAC1 Receptor
Source: Front Pharmacol. 2022 Jan 28;12:797541. doi: 10.3389/fphar.2021.797541 (PMC8832515; doi:10.3389/fphar.2021.797541)

FIGURE 3

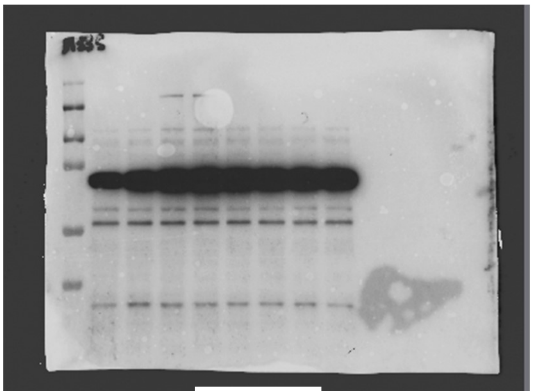

PAC1R

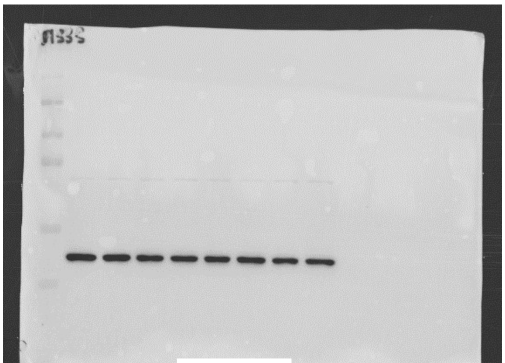

Actin

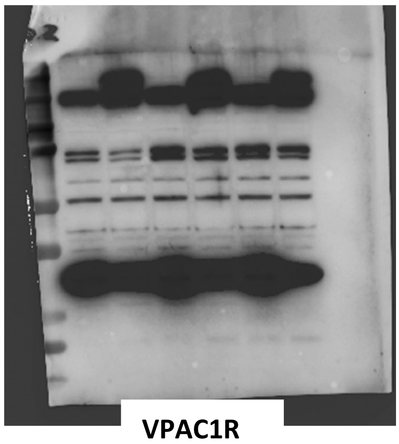

VPAC1R

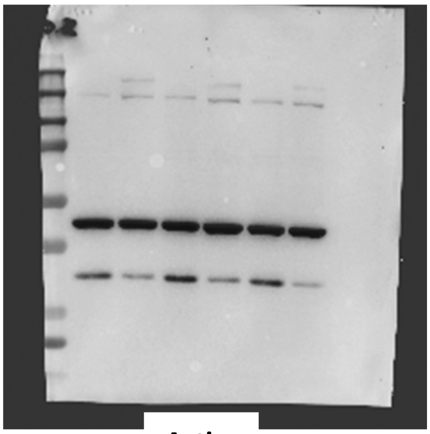

Actin

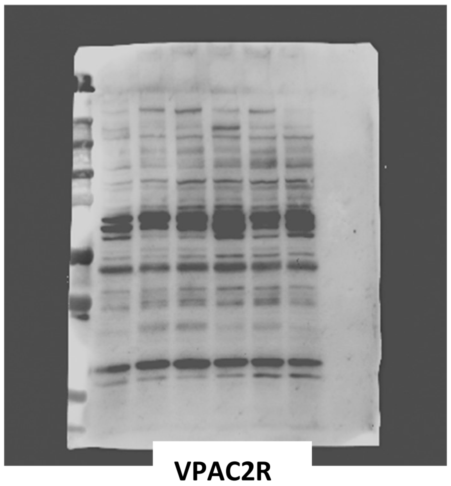

VPAC2R

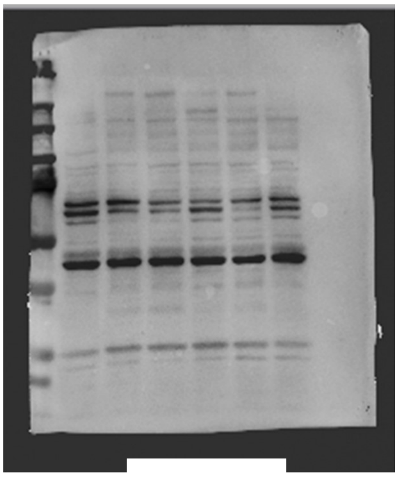

VPAC2R

FIGURE 4

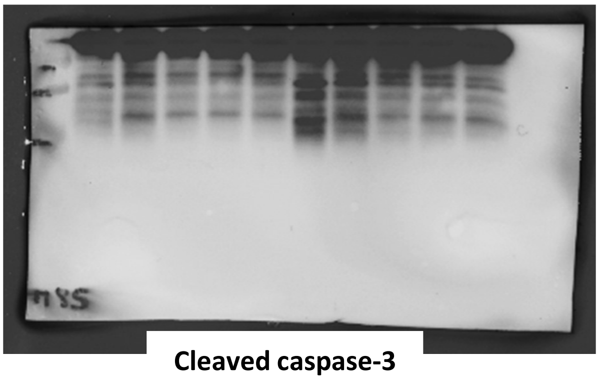

Cleaved caspase-3

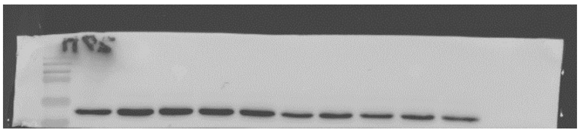

Actin

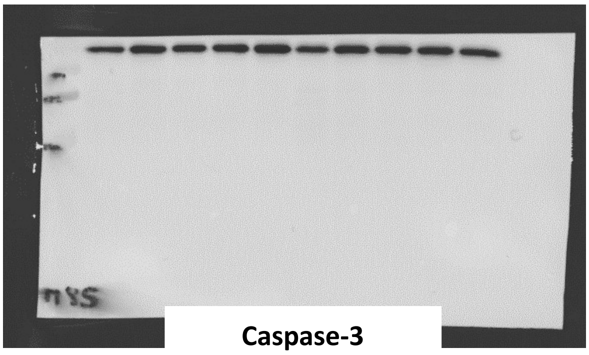

Caspase-3

FIGURE 5

A) PACAP- Q7

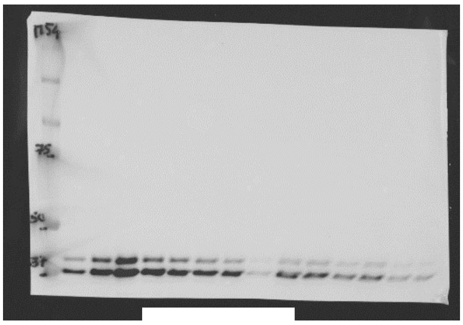

pERK

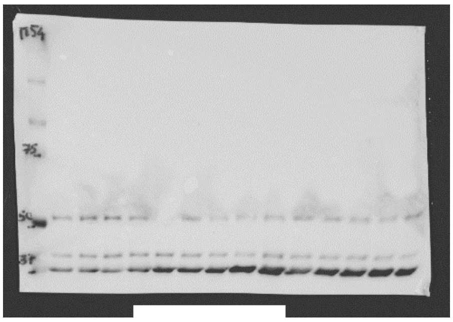

ERK

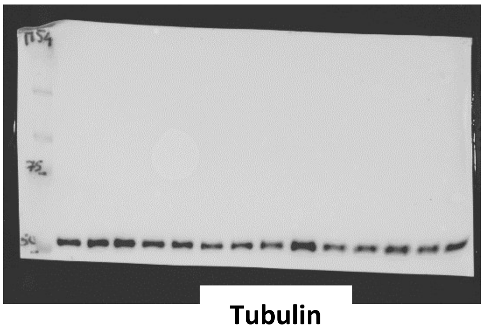

Tubulin

A) PACAP- Q111

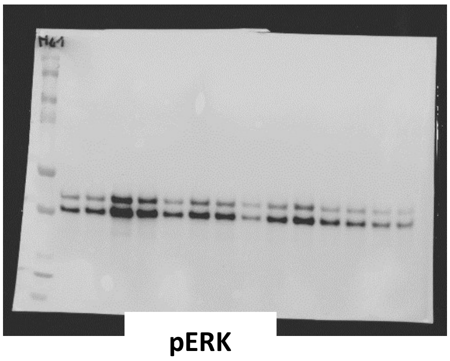

pERK

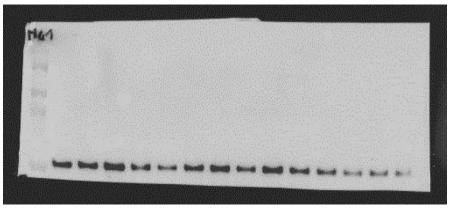

Tubulin

A) PACAP/ VIP 24H

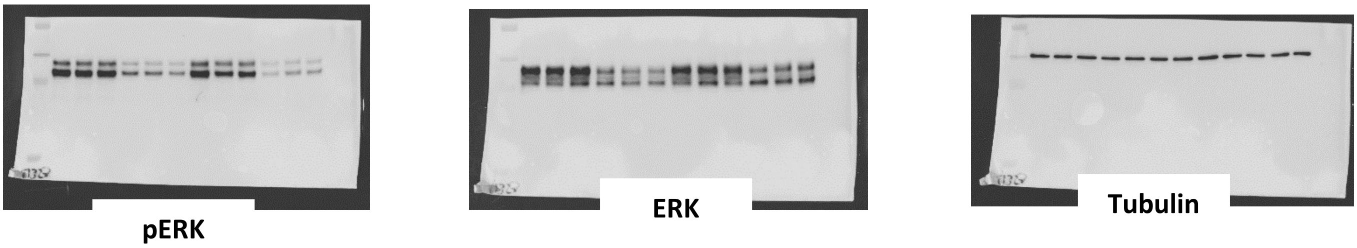

A) VIP

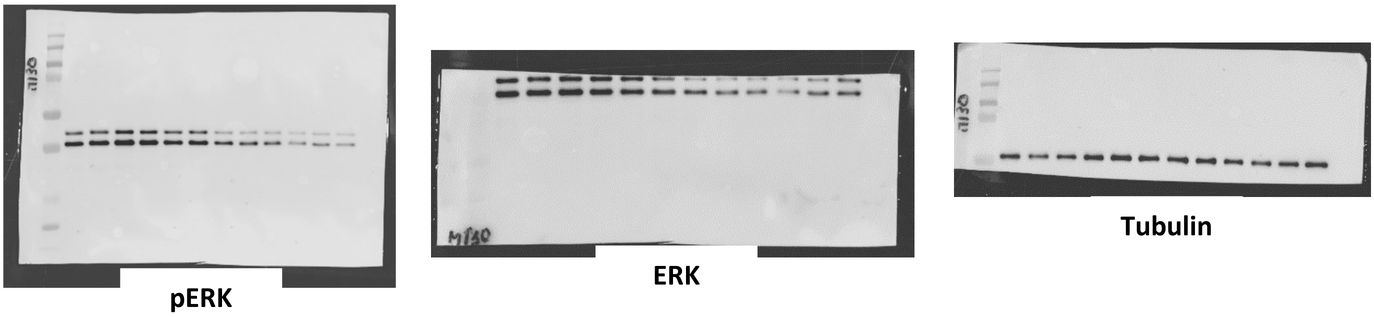

FIGURE 5

B) PACAP

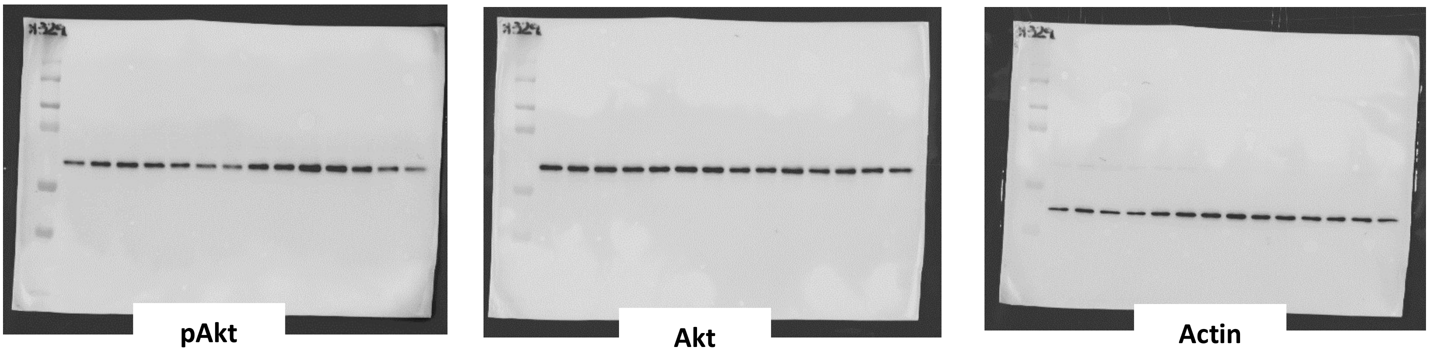

B) PACAP / VIP 24H

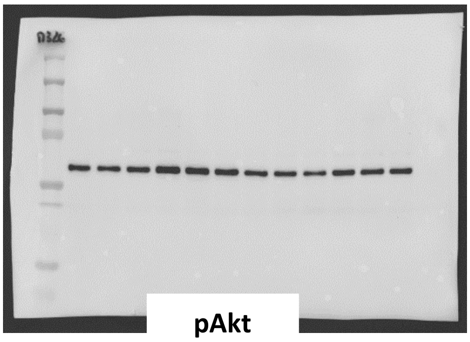

pAkt

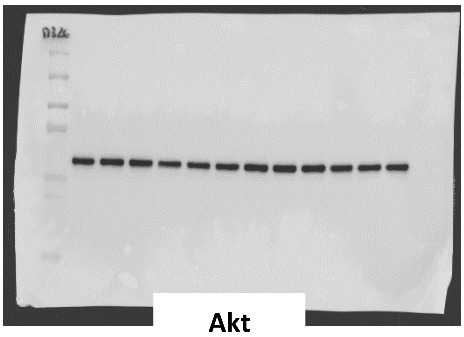

Akt

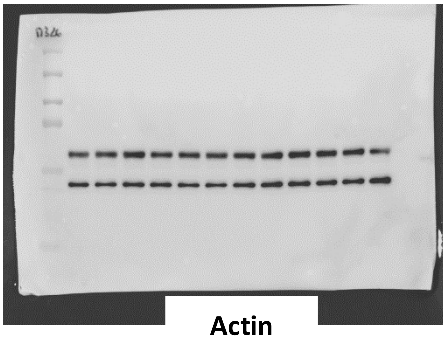

Actin

B) VIP

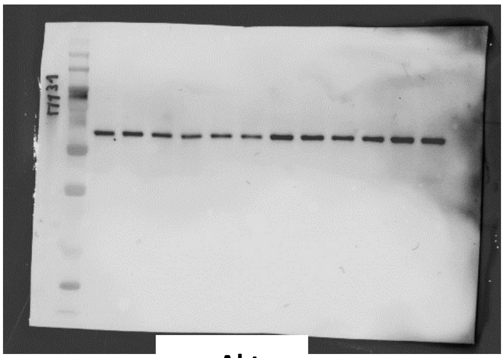

pAkt

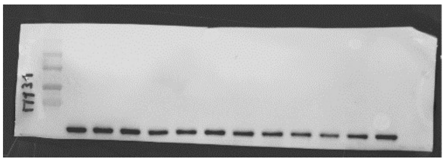

Akt

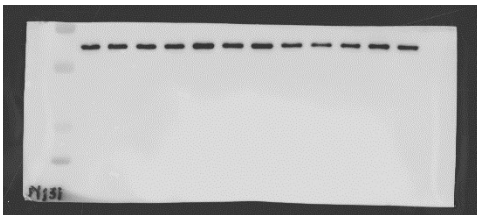

Actin

FIGURE 6

B)

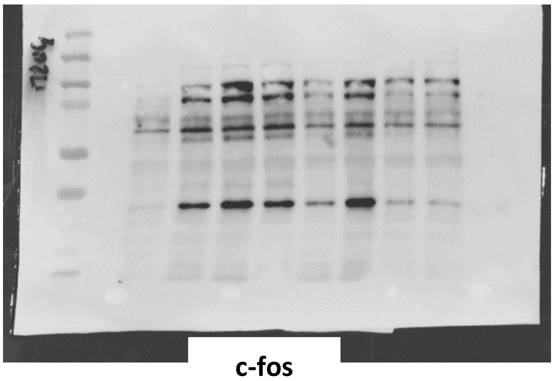

c-fos

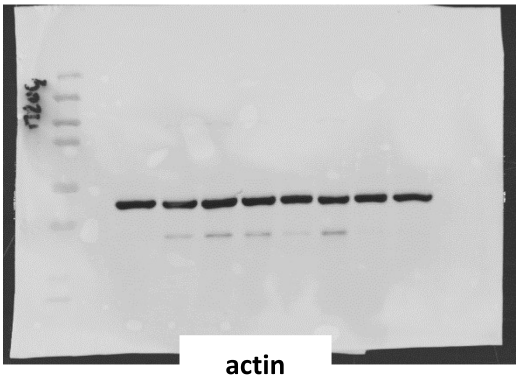

actin

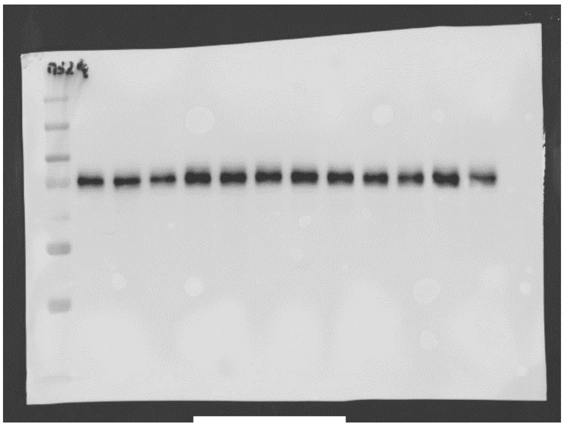

Egr-1

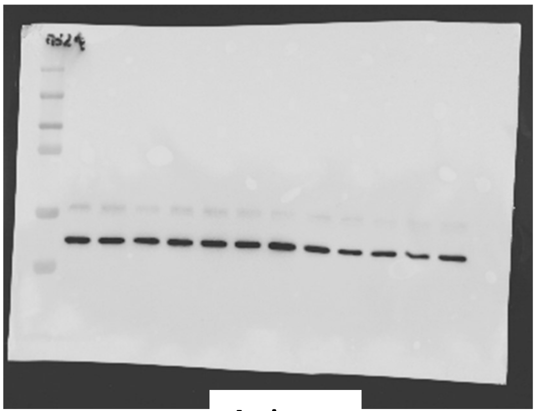

Actin

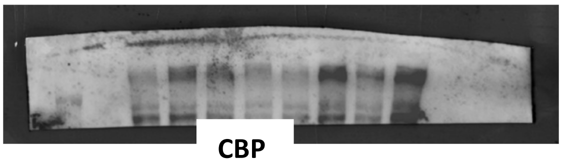

CBP

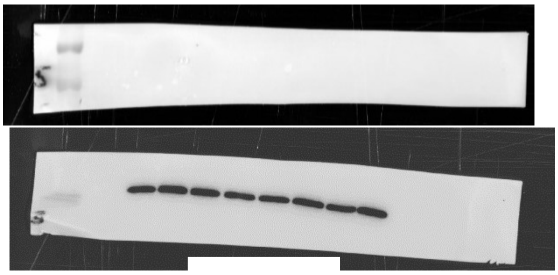

Tubulin

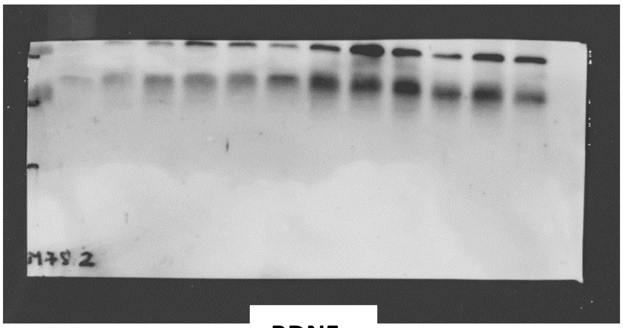

BDNF

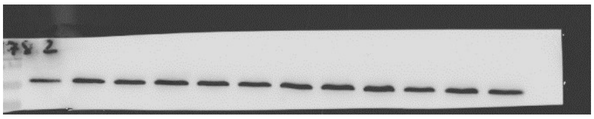

Tubulin

FIGURE 7

A) Q7

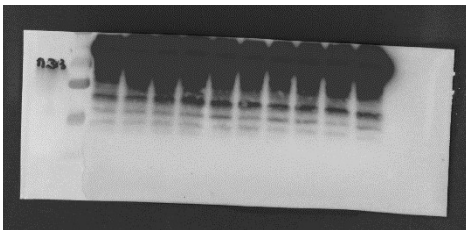

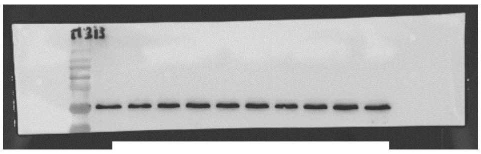

Tubulin

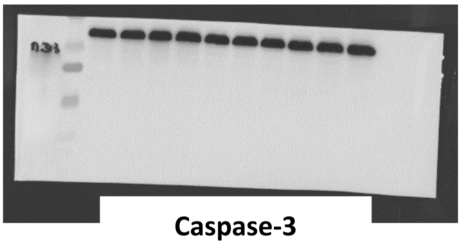

Cleaved caspase-3

Caspase-3

A) Q111

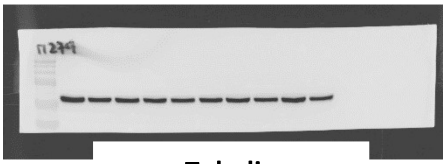

Tubulin

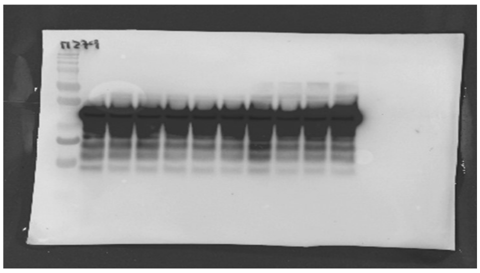

Cleaved caspase-3

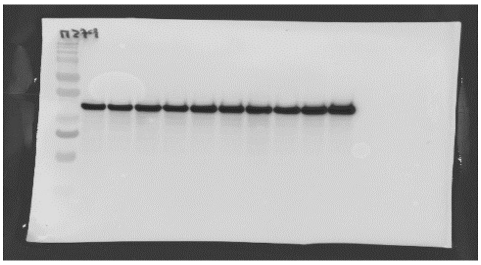

Caspase-3

B) Q7

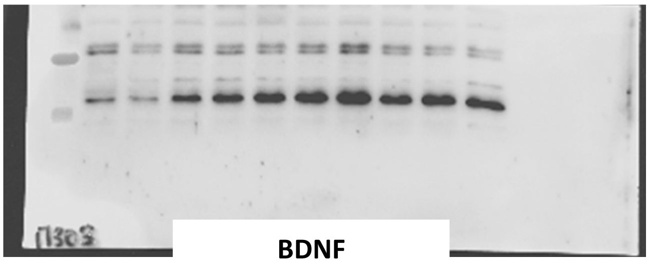

BDNF

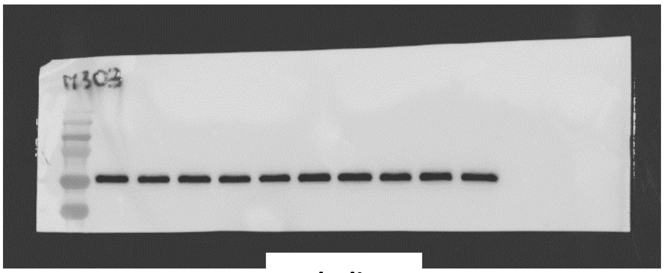

Tubulin

B) Q111

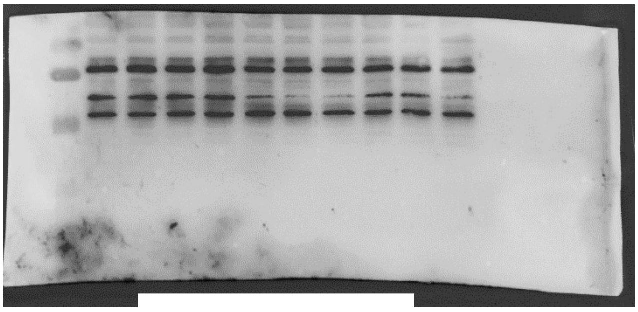

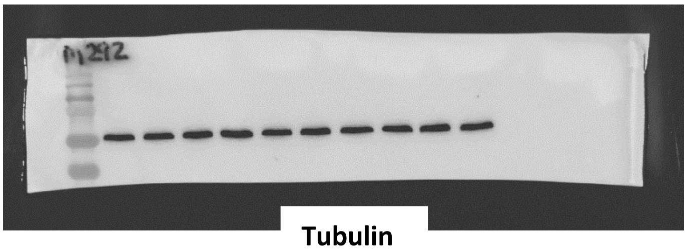

Tubulin

BDNF

FIGURE 8

<u>PAC1</u>

8 and 12 weeks

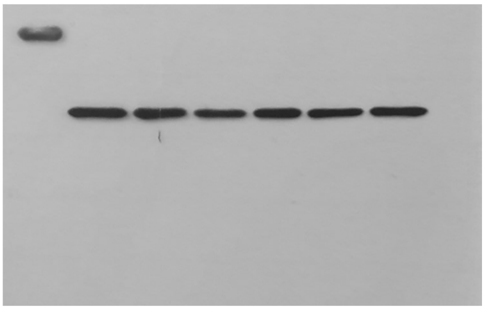

20 and 30 weeks

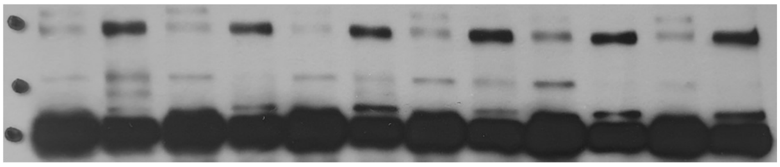

<u>VPAC1</u>

All ages

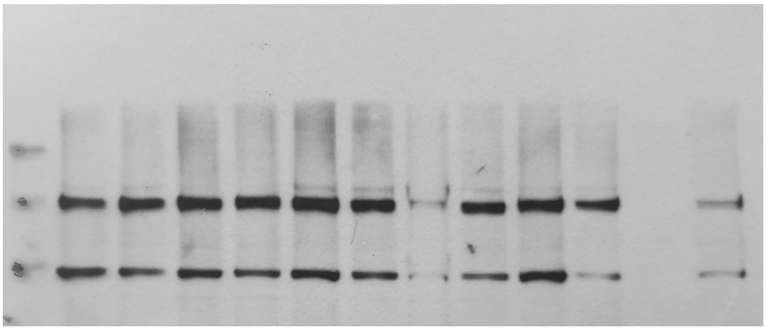

<u>VPAC2</u>

all ages

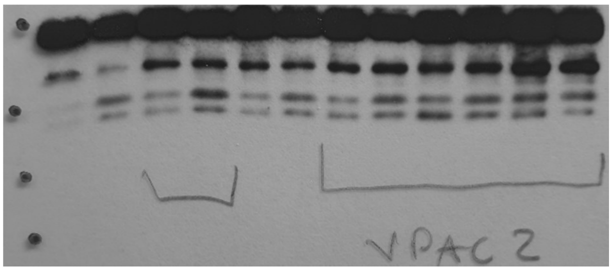

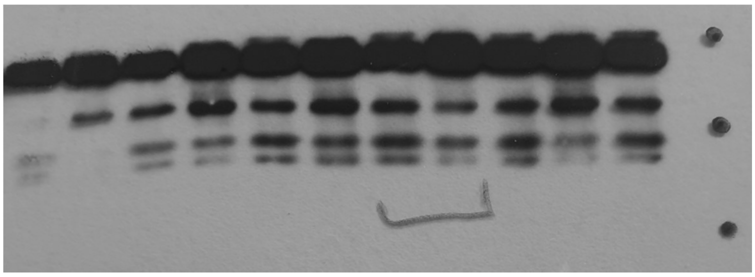

FIGURE 9:

<u>PAC1:</u> all ages

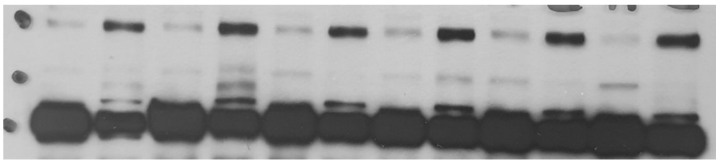

<u>VPAC1:</u> all ages

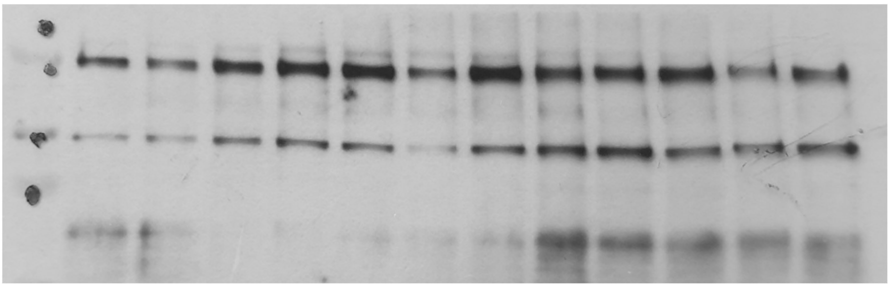

<u>VPAC2:</u> all ages

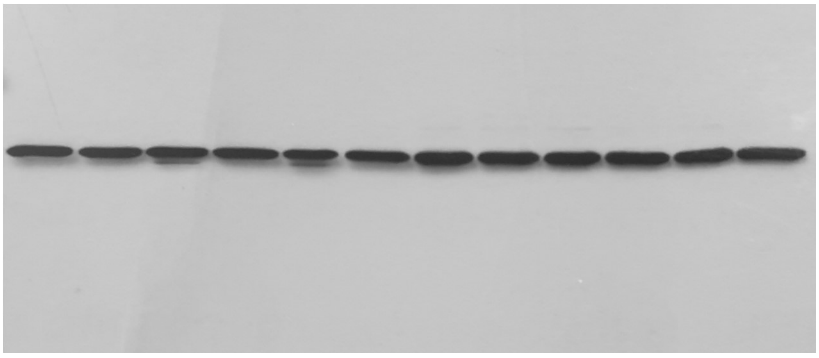

FIGURE 11:

PAC1:

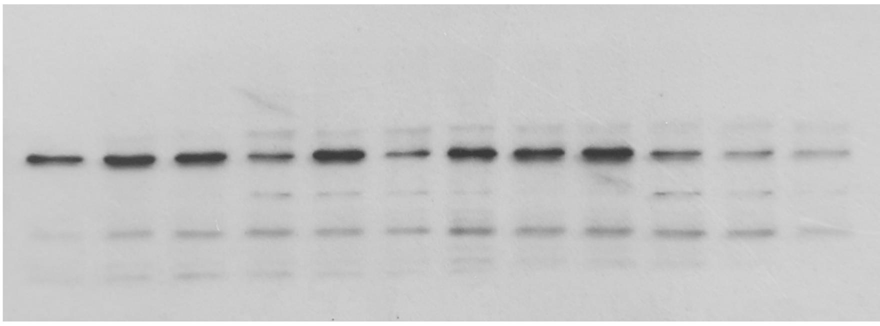

CBP:

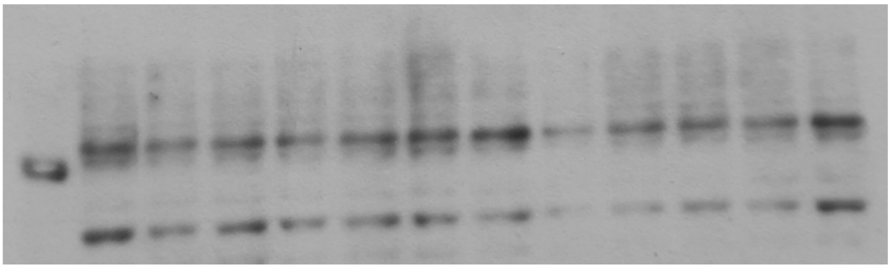

BDNF:

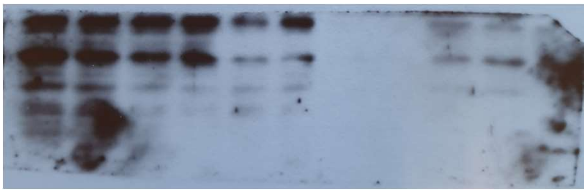

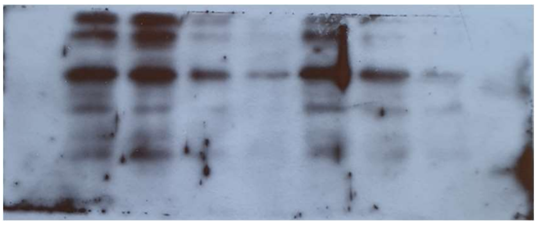

FIGURES 8, 9 AND 11

Tubulin: mixed samples

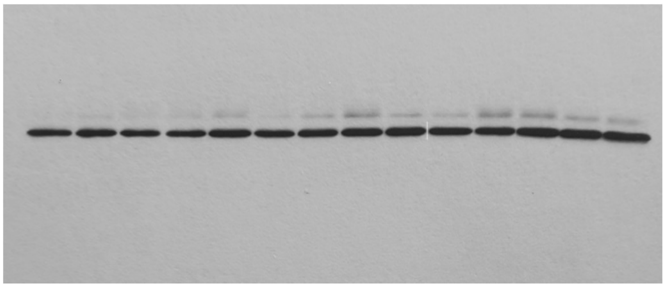

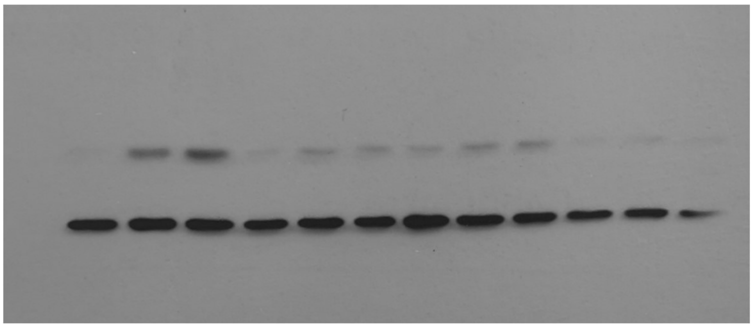

Supplement: Supplementary file 1 [file DataSheet1.PDF]
